# Multimodal regulation of the osteoclastogenesis process by secreted group IIA phospholipase A_2_

**DOI:** 10.3389/fcell.2022.966950

**Published:** 2022-08-29

**Authors:** Maria Mangini, Rosa D’Angelo, Caterina Vinciguerra, Christine Payré, Gérard Lambeau, Barbara Balestrieri, Julia F. Charles, Stefania Mariggiò

**Affiliations:** ^1^ Institute of Protein Biochemistry, National Research Council, Naples, Italy; ^2^ Institute of Biochemistry and Cell Biology, National Research Council, Naples, Italy; ^3^ Centre National de la Recherche Scientifique, Institut de Pharmacologie Moléculaire et Cellulaire, Université Côte d’Azur, Valbonne Sophia Antipolis, France; ^4^ Jeff and Penny Vinik Center for Translational Immunology Research, Department of Medicine, Division of Allergy and Clinical Immunology, Brigham and Women’s Hospital, Harvard Medical School, Boston, MA, United States; ^5^ Departments of Orthopaedic Surgery and Medicine, Brigham and Women’s Hospital, Harvard Medical School, Boston, MA, United States

**Keywords:** group IIA secreted phospholipase A_2_ (sPLA_2_-IIA), p38 SAPK, osteoclastogenesis, osteoclast syncytia, LLKYK cyclic peptide

## Abstract

Increasing evidence points to the involvement of group IIA secreted phospholipase A_2_ (sPLA_2_-IIA) in pathologies characterized by abnormal osteoclast bone-resorption activity. Here, the role of this moonlighting protein has been deepened in the osteoclastogenesis process driven by the RANKL cytokine in RAW264.7 macrophages and bone-marrow derived precursor cells from BALB/cJ mice. Inhibitors with distinct selectivity toward sPLA_2_-IIA activities and recombinant sPLA_2_-IIA (wild-type or catalytically inactive forms, full-length or partial protein sequences) were instrumental to dissect out sPLA_2_-IIA function, in conjunction with reduction of sPLA_2_-IIA expression using small-interfering-RNAs and precursor cells from *Pla2g2a* knock-out mice. The reported data indicate sPLA_2_-IIA participation in murine osteoclast maturation, control of syncytium formation and resorbing activity, by mechanisms that may be both catalytically dependent and independent. Of note, these studies provide a more complete understanding of the still enigmatic osteoclast multinucleation process, a crucial step for bone-resorbing activity, uncovering the role of sPLA_2_-IIA interaction with a still unidentified receptor to regulate osteoclast fusion through p38 SAPK activation. This could pave the way for the design of specific inhibitors of sPLA_2_-IIA binding to interacting partners implicated in osteoclast syncytium formation.

## 1 Introduction

Among the lipolytic enzymes, phospholipases A_2_ (PLA_2_) are acyl esterases, originally defined by their positional specificity to cleave the *sn-2* ester bond of glycerophospholipids, to release fatty acids and lysophospholipids (Dennis, 1994). Currently, more than 50 mammalian proteins are classified within the PLA_2_ family, based on the homology with prototypical enzymes, even if they do not necessarily exert a PLA_2_ activity (Murakami et al., 2020). Based on their structural relationships, PLA_2_s are classified into secreted (Lambeau and Gelb, 2008), cytosolic (Kita et al., 2019), calcium-independent PLA_2_s (Ramanadham et al., 2015), and within other groups that include platelet-activating factor acetylhydrolase (Kono and Arai, 2019), lysosomal PLA_2_ (Shayman and Tesmer, 2019), PLA/acyltransferase (Hussain et al., 2017), α/β hydrolase (Thomas et al., 2014), and glycosylphosphatidylinositol-specific PLA_2_ subfamilies (Murakami et al., 2020).

In mammals, PLA_2_s participate in almost all biological processes, regulating pathophysiological functions related to membrane homeostasis, energy production and generation of body surface barriers, among others (Murakami, 2019). The peculiar tissue and cellular distribution of the different PLA_2_s (Murakami et al., 2017), in conjunction with their substrate specificities toward the fatty acyl and/or headgroup moieties of the membrane phospholipids (Astudillo et al., 2019), and the availability of functional cofactors or regulatory proteins (Murakami et al., 2020), ensures a variety of distinct functions among these enzymes (Murakami, 2019). The recent advent of lipidomics, combined with studies of gene-manipulated mice or human diseases caused by mutations of PLA_2_ enzymes, supported the definition of *in-vivo* functions of individual PLA_2_s (Murakami et al., 2010; Murakami et al., 2015; Yamamoto et al., 2017; Kita et al., 2019; Murakami et al., 2019; Murakami et al., 2020; Sun et al., 2021).

The subfamily of secreted (s)PLA_2_s comprises 12 mammalian proteins, namely IB, IIA, IIC (present in mice and rats, but pseudogene in humans; Tischfield et al., 1996), IID, IIE, IIF, III, V, X, XIIA, XIIB (catalytically inactive), and otoconin-95 (catalytically inactive structural protein in the inner ear; Lambeau and Gelb, 2008). Secreted PLA_2_s are typically characterized by a low molecular mass (13–18 kDa), a conserved three-dimensional helical structure with high disulfide bond content, and a calcium-dependent enzymatic activity through the conserved His-Asp catalytic dyad (Lambeau and Gelb, 2008; Murakami et al., 2010; Murakami et al., 2020). The most studied member, sPLA_2_-IIA, has a demonstrated primary function in host defense against invading pathogens (Doré and Boilard, 2019; Scott et al., 2021; Miki et al., 2022). As a secreted lipolytic enzyme, sPLA_2_-IIA shows bactericidal activity through hydrolysis of Gram-positive membranes (Weinrauch et al., 1998), and anti-parasitic function through hydrolysis of oxidized phospholipids in the plasma lipoproteins of malaria patients, thus promoting P*lasmodium falciparum* killing effect (Dacheux et al., 2019; Dacheux et al., 2021). Secreted PLA_2_-IIA is also referred as an inflammatory enzyme and amplifier of innate immune responses (Doré and Boilard, 2019; Doré et al., 2022). Indeed, by its hydrolysis of specific phospholipid substrates present in extracellular vesicles released by immune cells (Fourcade et al., 1995; Papadopoulos et al., 2020), or in extracellular mitochondria released by damaged organs or tissues, sPLA_2_-IIA generates lysophospholipids and non-esterified fatty acids, among which are eicosanoid precursors (Murakami et al., 2020; Kudo et al., 2022).

Secreted PLA_2_-IIA acts prevalently on extracellular lipids, and its membrane binding preference (phosphatidylglycerol > phosphatidylserine > phosphatidylcholine) suggests that it is almost inactive toward the intact plasma membrane of mammalian cells (Snitko et al., 1997; Koduri et al., 1998; Singer et al., 2002; Doré and Boilard, 2019). Loss of membrane asymmetry, due to glycerophospholipid scrambling, has been demonstrated during apoptosis (Atsumi et al., 1997), blood platelet activation (Suzuki et al., 2010), viral infection (Arganaraz et al., 2020; Snider et al., 2021), and the osteoclastogenesis of bone marrow-derived cells (Kang et al., 2020). This process can render the plasma membranes of mammalian cells susceptible to sPLA_2_-IIA attack (Olson et al., 2010).

The sPLA_2_-IIA cellular functions account for the enzymatic hydrolysis of glycerophospholipids, but can also occur in a catalysis-independent manner (Murakami et al., 2017; Doré and Boilard, 2019; Scott et al., 2021; Ivanusec et al., 2022). Several cellular and soluble receptors for sPLA_2_-IIA have been characterized until now, starting from the cell surface M-type receptor for secreted phospholipases (Cupillard et al., 1999; Lambeau and Lazdunski, 1999), and these may be involved in the activation of downstream cellular signaling pathways with hydrolytic activity-independent mechanisms (Saegusa et al., 2008; Ivanusec et al., 2022). On the other hand, the sPLA_2_-IIA protein is highly basic and can easily associate to the cell surface via heparin sulfated proteoglycans (Koduri et al., 1998; Murakami et al., 1999; Boilard et al., 2003; Boilard et al., 2006), and its interaction with the cellular membranes has been proposed to be sufficient to alter the lipid bilayer structure with consequent pathophysiological functions (Cirino et al., 1994; Lomonte et al., 1995).

Several studies have related sPLA_2_-IIA to bone metabolism, mainly under pathological conditions. Abnormal osteoclast activity is the primary cause of the pathophysiology of Paget’s disease of bone, for which pharmacological inhibition of osteoclast cell-to-cell fusion remains the best therapeutic strategy to mitigate bone loss and related fractures associated with this disorder (Coulthard et al., 2011; Ferraz-de-Souza and Correa, 2013). De Brum-Fernandes and collaborators showed that sPLA_2_-IIA is highly expressed in bones with high turnover, and in particular in Pagetic bone samples, and that sPLA_2_-IIA inhibition abrogates osteoclast differentiation and function (Allard-Chamard et al., 2014b). Other groups found that sPLA_2_-IIA inhibition prevents bone loss after ovariectomy in rats (Gregory et al., 2006), as well as bone erosion in rheumatoid arthritis (Bradley et al., 2005; Thwin et al., 2009). However, the precise role of sPLA_2_-IIA in osteoclast formation and activity remained to be determined, as well as the role of its metabolites.

Here we provide evidence of a multimodal regulation of the osteoclastogenesis and of mature osteoclast activity by sPLA_2_-IIA, using RAW264.7 murine macrophages as osteoclast precursors, or primary precursor cells from bone marrow of wild-type (wt) and *Pla2g2a*-knockout (ko) BALB/cJ mice. We also collected insights about the catalytically independent participation of sPLA_2_-IIA in the selective regulation of osteoclast cell-to-cell fusion, through the activation of p38 SAPK-dependent pathways.

## 2 Materials and methods

### 2.1 Materials

Dulbecco’s modified Eagle’s medium (DMEM) supplemented with high glucose and GlutaMAX, Eagle’s alpha-modified medium (α-MEM) supplemented with GlutaMAX, fetal bovine serum (cod. 10270), Hank’s balanced salt solution supplemented without (HBSS^−^) or with calcium and magnesium (HBSS^++^), and phosphate-buffered saline (PBS) were from Gibco (Life Technologies Italia, Monza, Italy). Ammonium chloride (NH_4_Cl), β-glycerophosphate, bovine serum albumin, Hoechst, l-glutamine, penicillin–streptomycin, pyrogallol, potassium bicarbonate (KHCO_3_), saponin, silver nitrate, sodium azide, sodium deoxycholate, sodium dodecyl sulphate, sodium fluoride, sodium orthovanadate, Triton X-100, Tween-20, Mowiol 4–88, bromoenol lactone (BEL, cod. B1552), cPLA_2_α inhibitor (cPLA_2_α–Inh., cod. 525143), SB203580 (cod. 559389), sPLA2-IIA Inhibitor I (Inhib-I, cod. 525145), KH064 (sPLA2 inhibitor, cod. S3319; or sPLA2-IIA inhibitor II, cod. 525146-M), LY311727 (cod. L6795), 2,4′-dibromoacetophenone (BPB, p-bromophenacyl bromide, cod. D38308), trifluoroacetic acid (TFA, cod. 91707) were from Sigma-Aldrich (St. Louis, MO, United States). Methyl arachidonyl fluorophosphonate (MAFP, cod. Ab141763) was from Abcam (Cambridge, UK). The purified recombinant mouse (mGIIA) and human sPLA_2_-IIA (hGIIA), the human sPLA_2_-IIA H48Q and mouse group V sPLA_2_ (mGV) (Edwards et al., 2002; Ghomashchi et al., 2017), as well as the control and anti‒mouse sPLA_2_-IIA IgG fractions were produced as described (Nevalainen et al., 2005; Eerola et al., 2006). All sPLA_2_ preparations had a purity higher than 99%, and were routinely checked for LPS contamination (Limulus amebocyte test, MP Biomedicals, Illkirch, France; or other sensitive assays), and discarded if the LPS concentration was above the detection limit of the assay (0.125 EU/mL). The synthetic cyclic peptides: FLSYK, WDIYR, LLKYK, with an amide bond between N- and C-terminus, were synthesized with a purity higher than 98.8% from Caslo ApS (Lyngby, Denmark). All other reagents were obtained at the highest purities available from Merck Life Science (Milano, Italy).

### 2.2 Animals

All the experiments with animals were performed at the Brigham and Women’s Hospital, Harvard Medical School, Boston, MA, United States. The wild-type BALB/cJ mice were purchased from the Jackson Laboratory (Bar Harbor, ME, United States). The *Pla2g2a*-ko mice with a BALB/cJ genetic background were generated as previously described (Balestrieri et al., 2006), and backcrossed for 11 generations to a BALB/cJ background (Boilard et al., 2010). All the animal procedures were approved by the Institutional Animal Care and Use Committee for Brigham and Women’s Hospital, and conformed to relevant guidelines and laws.

### 2.3 Cell culture

The RAW264.7 murine monocytes/macrophages (cod. TIB-71) were from American Type Culture Collection (ATCC, Manassas, VA, United States), and cultured in DMEM with 10% heat-inactivated (30 min at 55°C) fetal bovine serum, 2 mM l-glutamine, 100 U/mL penicillin and 100 μg/mL streptomycin. RAW264.7 cells were used between passages 10 and 30.

For transient interference, RAW264.7 cells were plated at 300,000 cells/well in 12-well plate in 1 mL growth medium without antibiotics. After 24 and 48 h, cells were double transfected with 125 pmol/well of small-interfering (si)RNAs against sPLA_2_-IIA (si–sPLA_2_-IIA) (murine group II sPLA_2_ siRNA, sc-43818, from Santa Cruz Biotecnology, San Diego, CA, United States), or with non-targeting siRNAs (si–NT) (siGENOME non-targeting siRNA pool #2, cat. No. D-001206–14 from Dharmacon, Chicago, IL, United States), using Lipofectamine RNAiMAX reagent (Invitrogen, Carlsbad, CA, United States), according to the manufacturer instructions. One day after the second interference, cells were detached (with 600 μM EDTA in PBS), and plated for the *in-vitro* osteoclastogenesis assays (as reported below), or for RNA extraction to determine the interference efficiency at 72 h of siRNA treatment.

### 2.4 Osteoclastogenesis assays

RAW264.7 cells were plated in differentiation medium (α-MEM without nucleosides supplemented with 10% heat-inactivated fetal bovine serum, 100 U/mL penicillin, and 100 μg/mL streptomycin) at a density of 1,250 cells/well in 96-well plates; at 5,000 cells/well on coverslips in 24-well plates for morphological analysis; or at 10,000 cells/well in 12-well plates for RNA extraction. Twenty-four hours later, and after every 48 h, the medium was replaced, and the cells were treated without or with 15–30 ng/mL ‘Receptor activator of nuclear factor kappa-Β ligand’ (RANKL) (cod. 310–01 from Peprotech, London, UK), in the absence or presence of different agents diluted in the differentiation medium. The RANKL concentration used was adjusted according to batch-to-batch variability of the cytokine. The range of RANKL concentration specified in several figure/table legends implies that all the different treatments shown were analyzed under the same differentiation conditions of control samples. When differentiation was complete, cells were harvested in lysis buffer for RNA extraction or processed for the different assays (as reported below).

The *ex-vivo* osteoclastogenesis assays were carried out at the Brigham and Women’s Hospital, according to published protocols (Charles et al., 2012; O'Brien et al., 2016). After euthanasia of mice, the femurs and tibias were removed and washed in 70% ethanol, and then with HBSS^−^. The bone ends were excised, and the bone marrow flushed out. The extracted bone marrow cells were collected by centrifugation. Subsequently, the red blood cells were lysed by 2-min treatment with lysis solution (0.15 M NH_4_Cl, 1 mM KHCO_3_, in water), and the reaction was stopped by addition of fetal bovine serum and subsequent centrifugation. Total bone marrow cells (BMC) were plated at a density of 50,000 cells/well in 96-well plates to be differentiated for 4 days in differentiation medium for primary cells (α-MEM with nucleosides supplemented with 10% heat-inactivated fetal bovine serum, 100 U/mL penicillin, and 100 μg/mL streptomycin), containing 20 ng/mL M-CSF and 5 ng/mL RANKL, and then fixed for TRAP staining. In parallel, BMC were treated with 40 ng/mL M-CSF for 4 days in α-MEM with nucleosides supplemented with 10% heat-inactivated fetal bovine serum, 100 U/mL penicillin, and 100 μg/mL streptomycin, to expand the osteoclast precursor cells (OPC). After expansion, adherent OPC were detached using the Gibco cell dissociation buffer, and were plated at 10,000 cells/well in 96-well plates for TRAP staining and degradation assays (on Osteo Assay Surface), or at 40,000 cells/well in 24-well plates for immunofluorescence analysis, or at 60,000 cells/well in 12-well plates for RNA extraction. These cells were treated with 20 ng/mL M-CSF without or with 2.5 ng/mL RANKL in differentiation medium for primary cells, and the medium was replenished every other day. Differentiation was usually complete after 3–6 days. At the end, the cells were fixed with 4% (w/v) paraformaldehyde for TRAP staining and immunofluorescence analysis, or harvested in lysis buffer for RNA extraction (as reported below).

### 2.5 Ribonucleic acid extraction and real-time quantitative polymerase chain reaction

Total RNA was extracted using RNeasy isolation kits, cDNAs were obtained using QuantiTect Reverse Transcription kits, and real-time qPCR was performed with QuantiTect SYBR Green PCR kits, all according to the manufacturer instructions (all the kits were from Qiagen, Hilden, Germany). The primers used for real-time qPCR (LightCycler 480 Instrument II; Roche, Indianapolis, IN, United States) are listed in Supplementary Table S1. *β*_*2*_ *-Microglobulin* was used as housekeeping gene. The real-time qPCR program consisted of an initial step of 15 min at 95°C, and then 45 cycles, as follows: 94°C for 15 s, annealing temperature (see Supplementary Table S1) of each primer for 30 s, and 72°C for 30 s. The osteoclastogenesis marker mRNA levels, in cells subjected to pharmacological treatments, were quantified by real-time qPCR as fold of control levels, i.e., mRNA levels in cells treated with RANKL alone.

### 2.6 Measurement of sPLA_2_ catalytic activity

The sPLA_2_ enzymatic activity was determined using a commercially available colorimetric kit, sPLA_2_ Assay kit from the Cayman Chemical Company (cod. 765001, Ann Arbor, MI, United States). Briefly, cells were scraped in assay buffer (25 mM Tris-HCl pH 7.5, 10 mM CaCl_2_, 100 mM KCl, 0.3 mM Triton X-100) and lysed by sonication. The protein concentration was determined using the Bradford assay, and 250 μg total cell lysate was used to determine the catalytic activity, following the kit manufacturer instructions.

### 2.7 Immunofluorescence microscopy

After treatment with or without RANKL, cells were rinsed with HBSS^++^ and fixed in 4% (w/v) paraformaldehyde (Electron Microscopy Sciences, Hatfield, PA, United States) for 10 min at room temperature, and then washed three times with HBSS^++^. Fixed cells were incubated for 1 h with blocking solution (50 mM NH_4_Cl, 0.5% bovine serum albumin, 0.1% saponin, 0.02% sodium azide, in PBS), with 33 nM Alexa546-labeled phalloidin (Invitrogen) for filamentous actin visualization, and 2 μg/mL Hoechst for nucleus staining. Finally, the cells were washed three times with PBS plus 0.02% Tween-20, and the coverslips were mounted with Mowiol 4–88 and examined by confocal microscopy (LSM 510; Zeiss, Oberkochen, Germany). For the evaluation of osteoclast syncytium formation, the nuclei of multinucleated cells were counted in a blinded manner using a 63× objective, moving across the coverslip in the vertical and horizontal directions. No evident differences in cell numbers, due to cell toxicity or changes in proliferation rates, were observed for the differentiated cells treated in the absence or presence of the different treatments. As Inhib-I was purchased as trifluoroacetate (TFA) salt, the absence of cell toxicity induced by TFA was verified treating RAW264.7 cells with 50 µM TFA for up to 120 h (with 3 additions and medium changes every 48 h) in differentiation medium (in absence of RANKL), and analyzing annexin-V staining (Annexin V-FITC kit, from Milteny Biotech GmbH, Germany), and propidium iodide incorporation by FACS analysis.

### 2.8 TRAP staining and degradation assay

For TRAP staining, fixed cells were incubated with staining solution (1.5% sodium tartrate, 0.1% naphthol AS-MX phosphate, 1% N,N-dimethylformamide, 0.6% Fast Red LB Violet Salt in 0.1 M acetate buffer) for 15 min at 37°C. The reaction was stopped by washing cells three times with HBSS^++^. Osteoclasts were quantified by counting TRAP-positive cells with three or more nuclei.

To measure resorptive function, osteoclasts were grown on Osteo Assay Surface plates from Corning (New York, United States) for 7 days. When differentiation was complete, the cells were removed (using 10% bleach) and the wells were stained with 5% silver nitrate for 1 h. The staining was stopped by addition of 1% pyrogallol. Images were taken with a Nikon TMS-F inverted microscope (Nikon Instruments Inc., Melville, NY, United States) at a final magnification of ×5 (BMC and OCP), or with EVOS XL Core microscope (Thermo Fisher Scientific, Waltham, MA United States) at a final magnification of ×2 (RAW264.7 derived osteoclasts), and the total resorbed areas were quantified using ImageJ (NIH).

### 2.9 Western blotting

Cell lysates were obtained by scraping cells into phospho-lysis buffer (50 mM Tris-HCl, pH 7.5, 300 mM NaCl, 1% sodium deoxycholate, 1% Triton X-100, 0.1% sodium dodecyl sulphate, 40 mM β-glycerophosphate, 50 mM sodium fluoride, 200 µM sodium orthovanadate, plus phosphatase and protease inhibitors from Roche). Following gentle homogenization by 20 passages through a 26-gauge needle, the lysates were centrifuged at 10,000× *g* for 5 min at 4°C, and the supernatants were collected.

Protein lysates were subjected to SDS-PAGE, and after electrophoresis, the proteins were transferred to a nitrocellulose membrane (PerkinElmer Life Science, Boston, MA, United States). For immunoblotting, the membranes were blocked with 5% non-fat dry milk in TBS (10 mM Tris-HCl, pH 7.4, 150 mM NaCl) plus 0.1% Tween-20 (T-TBS) for 30 min at room temperature, and incubated with primary antibodies (as listed below) in T-TBS plus 3% bovine serum albumin for 2 h at room temperature, or overnight at 4°C. The membranes were washed twice in T-TBS for 7 min, and then incubated with secondary antibodies conjugated to horseradish peroxidase (1:5,000) (Calbiochem, San Diego, CA, United States) in T-TBS with 5% non-fat milk for 30 min at room temperature. The membranes were then washed twice with T-TBS, and once with TBS for 5 min, and the signals were detected by ECL (Amersham Pharmacia, Piscataway, NJ, United States). The rabbit anti-phospho AKT (Ser473; D9E, cod. 4060), anti phospho-p44/42 MAPK (Thr202/Tyr204; Erk1/2, cod. 9101), anti-phospho p38 MAPK (Thr180/Tyr182; 3D7, cod. 9215), anti-p38 MAPK (cod. 9212), anti-phospho MAPKAPK-2 (T334; 27B7, cod. 3007), anti-MAPKAPK-2 (D1E11, cod. 12155), all used at dilution 1:1,000 were from Cell Signaling Technology (Danvers, MA, United States). The rabbit anti-AKT (B-1, cod. sc-5298, diluted 1:1,000), and anti-ERK1 (K-23, cod. sc-94, diluted 1:5,000) were from Santa Cruz Biotechnology.

### 2.10 Statistical analysis

Statistical analysis was performed with the GraphPad Prism software (GraphPad Software, Inc., La Jolla, CA, United States). Comparisons between groups were performed using Student’s *t*-test and Analysis of Variance (ANOVA) with 95% confidence interval. *p*  values < 0.05 were considered as statistically significant.

## 3 Results

### 3.1 Secreted PLA_2_-IIA mRNA levels and enzymatic activity increase upon RANKL-induced murine osteoclastogenesis

The osteoclastogenesis process was assessed with the RAW264.7 monocyte/macrophage cell line (Collin-Osdoby and Osdoby, 2012; Mosca et al., 2021) and with primary osteoclast precursors from bone marrow of BALB/cJ mice (Charles et al., 2012; O'Brien et al., 2016). ‘Receptor activator of nuclear factor kappa-Β ligand’ (RANKL) alone or in the presence of ‘macrophage colony-stimulating factor’ (M-CSF) was used to induce osteoclast differentiation of RAW264.7 cells or bone marrow-derived precursors, respectively.

Transcriptional analysis of most PLA_2_ family members, as assessed by real-time qPCR, indicated that the mRNA levels of sPLA_2_-IIA, -IIC, -IIE, and -V increased upon RANKL-induced differentiation of RAW264.7 cells, while that of sPLA_2_-IB, -III and of intracellular cPLA_2_α (PLA_2_-IVA) and iPLA_2_β (PLA_2_-VIA) decreased (Table 1). Other sPLA_2_s (-XIIA, -XIIB) and PLA_2_-XVI showed almost unchanged mRNA levels, while sPLA_2_-IID, -IIF, -X were not detectable (Table 1). These modulations were comparable to those observed within the osteoclastogenesis of bone marrow-derived M-CSF–expanded osteoclast precursors (OCP) from wt mice, with few exceptions: sPLA_2_-IIC was not detectable in the primary system, sPLA_2_-III was not modulated, and the iPLA_2_β levels increased during the osteoclastogenesis of wt OCP (Table 1; data from *Pla2g2a*-ko mice are commented in paragraph 3.3).

**TABLE 1:** Modulation of PLA_2_ mRNA levels during murine osteoclastogenesis. RAW264.7 cells were treated without (w/o) or with 15–30 ng/mL RANKL for 72 h, while M-CSF–expanded OCP from wild-type (wt) and *Pla2g2a*-ko mice were treated with 20 ng/mL M-CSF alone (M-CSF) or with 2.5 ng/mL RANKL (M-CSF+RANKL) for 3–6 days, before RNA extraction.

	RAW264.7 cells		wt OCP		*Pla2g2a*-ko OCP	
PLA2 gene (protein)	w/o	RANKL	M-CSF	M-CSF+ RANKL	M-CSF	M-CSF+ RANKL
*Pla2g1b* (sPLA_2_-IB)	1.00	0.68 ± 0.04*	1.00 ± 0.04	0.60 ± 0.03^§§^	1.44 ± 0.09^§§^	0.58 ± 0.03^††^
*Pla2g2a* (sPLA_2_-IIA)	1.00	2.51 ± 0.21*	1.00 ± 0.06	1.98 ± 0.02^§§§^	‒	‒
*Pla2g2*c (sPLA_2_-IIC)	1.00	4.27 ± 0.35*	‒	‒	‒	‒
*Pla2g2d* (sPLA_2_-IID)^**a**^	‒	‒	‒	‒	‒	‒
*Pla2g2e* (sPLA_2_-IIE)	1.00	3.95 ± 0.56*	1.00 ± 0.26	1.88 ± 0.15^§§^	3.25 ± 0.39^§§^	6.57 ± 0.83^††^
*Pla2g2f* (sPLA_2_-IIF)^**a**^	‒	‒	‒	‒	‒	‒
*Pla2g3* (sPLA_2_-III)	1.00	0.82 ± 0.02*	1.00 ± 0.10	1.06 ± 0.11	1.16 ± 0.13	1.12 ± 0.16
*Pla2g5* (sPLA_2_-V)	1.00	2.62 ± 0.21*	1.00 ± 0.07	2.96 ± 0.28^§§^	2.42 ± 0.21^§§§^	4.89 ± 0.40^††^
*Pla2g10* (sPLA_2_-X)^**b**^	‒	‒	‒	‒	‒	‒
*Pla2g12a* (sPLA_2_-XIIA)	1.00	1.08 ± 0.06	1.00 ± 0.02	0.99 ± 0.06	1.21 ± 0.18	0.87 ± 0.06
*Pla2g12b* (sPLA_2_-XIIB)	1.00	0.86 ± 0.05	1.00 ± 0.11	0.99 ± 0.10	0.88 ± 0.12	0.74 ± 0.04
*Pla2g4a* (PLA_2_-IVA, cPLA_2_α)	1.00	0.61 ± 0.02***	1.00 ± 0.05	0.56 ± 0.08^§^	1.24 ± 0.14	0.50 ± 0.06^†^
*Pla2g6* (PLA_2_-VIA, iPLA_2_β)	1.00	0.64 ± 0.02**	1.00 ± 0.08	1.59 ± 0.05^§^	0.95 ± 0.06	1.15 ± 0.17
*Pla2g16* (PLA_2_-XVI)^**c**^	1.00	0.91 ± 0.22	nd	nd	nd	nd

The transcripts were quantified by real-time qPCR, and normalized using *β2-microglobulin* expression, as the housekeeping gene. For RAW264.7 cells, PLA_2_ mRNA, levels are shown as fold of cells treated without RANKL (w/o), and data are means ± SEM, of at least three independent experiments. For OCP, PLA_2_ mRNA, levels are expressed as fold of wild-type OCP, treated only with M-CSF, and data are means ± SEM, of ten (sPLA_2_-IIA, sPLA_2_-V, sPLA_2_-IIE) or five (other PLA_2_s) age- and sex-matched mice for each genotype. *p < 0.05, **p < 0.005, ***p < 0.001 versus RAW264.7 cells treated without RANKL (w/o) (by paired Student’s *t*-tests). ^§^ *p* < 0.05, ^§§^ *p* < 0.005, ^§§§^ *p* < 0.001 versus wt OCP, treated with M-CSF alone; and ^†^ *p* < 0.05, ^††^ *p* < 0.005 versus ko OCP, treated with M-CSF alone; by one-way ANOVA). nd, not determined; ‒, undetectable, with primer efficiency verified with cDNA, of RAW264.7 cells treated for 1 h with 100 ng/mL lipopolysaccharide in DMEM, as reported in (Ao et al., 2010) (^**a**^), or with testis cDNA (^**b**^). *Pla2g16* transcription was analyzed in RAW264.7 cells treated with a single concentration of RANKL (15 ng/mL) (^**c**^).

Phospholipase A_2_ activity was assayed in cell extracts from undifferentiated or RANKL-treated RAW264.7 macrophages with a commercially available kit (see Section 2 for details), based on the use of the 1,2-dithio analog of diheptanoyl phosphatidylcholine, which serves as a substrate for most PLA_2_s (Hendrickson et al., 1983; Reynolds et al., 1992). A 6-fold increase in PLA_2_ activity was measured in osteoclasts compared to undifferentiated cells (Table 2). This activity was not blocked by inhibitors of intracellular PLA_2_s, like the cPLA_2_α specific inhibitor (cPLA_2_α–Inh.; San Pietro et al., 2009; Seno et al., 2000); or the iPLA_2_β irreversible inhibitor (BEL; Ackermann et al., 1995; Zizza et al., 2012). Instead, various inhibitors of sPLA_2_s were efficient. Indeed, Inhib-I (Church et al., 2001; Zizza et al., 2012) reduced by 40%, while KH064 (Hansford et al., 2003; Kim et al., 2020) and the potent and covalent inhibitor of sPLA_2_-IIA and other sPLA_2_s (BPB; Kim et al., 2020; Marchi-Salvador et al., 2009) almost abolished the measured activity of RANKL lysates (Table 2). Moreover, a significant inhibition was seen with 10 μM LY311727, a quite specific inhibitor of sPLA_2_-IIA versus other mouse and human sPLA_2_s (Singer et al., 2002; Kim et al., 2020), which showed an apparent IC_50_ of 6.8 μM, and by a specific anti-mouse sPLA_2_-IIA IgG fraction (Eerola et al., 2006) (Table 2, Supplementary Figure S1).

**TABLE 2:** Secreted PLA_2_ activity measured in cell lysates. RAW264.7 cells were treated without (w/o) or with 15 ng/mL RANKL for 72 h, in the absence or presence of 10 nM BPB. Phospholipase A_2_ activity was analyzed in cell lysates (250 μg) in absence (‒) or presence of the indicated PLA_2_ inhibitors.

RAW264.7-cell treatment	PLA_2_ inhibitor	sPLA2 activity (nmoles/min/mL)
w/o	‒	0.40 ± 0.12**
	DMSO	0.43 ± 0.15^§^
	+10 μM LY311727	0.54 ± 0.17
RANKL	‒	2.51 ± 0.42
	DMSO	2.32 ± 0.50
	+2 μM cPLA_2_α–Inh.	3.24 ± 1.24^§^
	+1 μM BEL	2.33 ± 1.01
	+20 μM Inhib-I	1.38 ± 0.73^§^
	+40 μM KH064	0.09 ± 0.03^§^
	+100 nM BPB	0.05 ± 0.03^§^
	+10 μM LY311727	1.31 ± 0.34^§^
	+3 μg/assay ctrl IgGs	2.64 ± 0.02
	+3 μg/assay anti-mouse sPLA_2_-IIA IgGs	2.08 ± 0.01^††^
RANKL + BPB	‒	2.19 ± 0.44*

Data are means ± SEM, of at least three independent experiments. *p < 0.05, **p < 0.005 versus (‒) untreated, ^§^ *p* < 0.05 versus DMSO-treated, and ^††^ *p* < 0.005 versus ctrl-IgG-treated RANKL-differentiated RAW264.7 cell lysates (paired Student’s *t*-tests).

The monitored catalytic activity, together with the expression profile in the above settings, was suggestive of a sPLA_2_ role in RANKL-induced differentiation of murine precursor cells, reasonably that of sPLA_2_-IIA.

### 3.2 Secreted PLA_2_s mediate the murine osteoclastogenesis

In addition to sPLA_2_s (Bradley et al., 2005; Gregory et al., 2006; Allard-Chamard et al., 2014b), cytosolic PLA_2_s were reported to participate in the osteoclastogenesis process (Bonventre, 2004; Kim et al., 2010; Allard-Chamard et al., 2014a). Therefore, inhibitors targeting the three major types of PLA_2_s were used to unveil their role (if any) in the osteoclastogenesis process of RAW264.7 macrophages. Real-time qPCR analysis of the osteoclastogenesis markers showed that the sPLA_2_ inhibitor (Inhib-I) was efficient to reduce the RANKL-induced transcription of all the analyzed markers (*Nfatc1*, *Cathepsin-k*, *Mmp-9*, *Trap* and *Ctr*) by at least 50%, and was the only treatment able to significantly impair the osteoclast syncytium formation (Supplementary Figures S2, S3). Specific cPLA_2_α inhibition, by cPLA_2_α–Inh., reduced by 40% only *Nfatc1* mRNA levels, while iPLA_2_β blockage with BEL reduced *Nfatc1* and *Trap* mRNA levels by 35% and 45%, respectively, in RANKL-differentiated cells (Supplementary Figure S2). Instead, the active site directed, irreversible inhibitor of both cPLA_2_α and iPLA_2_β (MAFP; Balsinde and Dennis, 1996; Lio et al., 1996), was not able to inhibit the RANKL-induced transcription of any marker nor the osteoclast fusion (Supplementary Figures S2, S3).

Although long-term treatment of cells with pharmacological inhibitors has to be taken with extreme caution, these data supported the hypothesis of sPLA_2_ involvement, seemingly sPLA_2_-IIA, in the regulation of the osteoclastogenesis of RAW264.7 macrophages. Instead, intracellular PLA_2_s did not seem to play a relevant role, as their inhibitors mainly reduced RANKL-induced transcription of *Nfatc1*, while the other marker mRNA levels and the osteoclast-syncytium formation were almost comparable to that of cells treated with RANKL alone. The absence of any effect by the less specific inhibitor MAFP could be consequent to the blockade of both positive and negative pathways regulated by distinct PLA_2_ isoforms.

### 3.3 Secreted PLA_2_-IIA plays a role in murine osteoclast differentiation and activity

**FIGURE 1:**
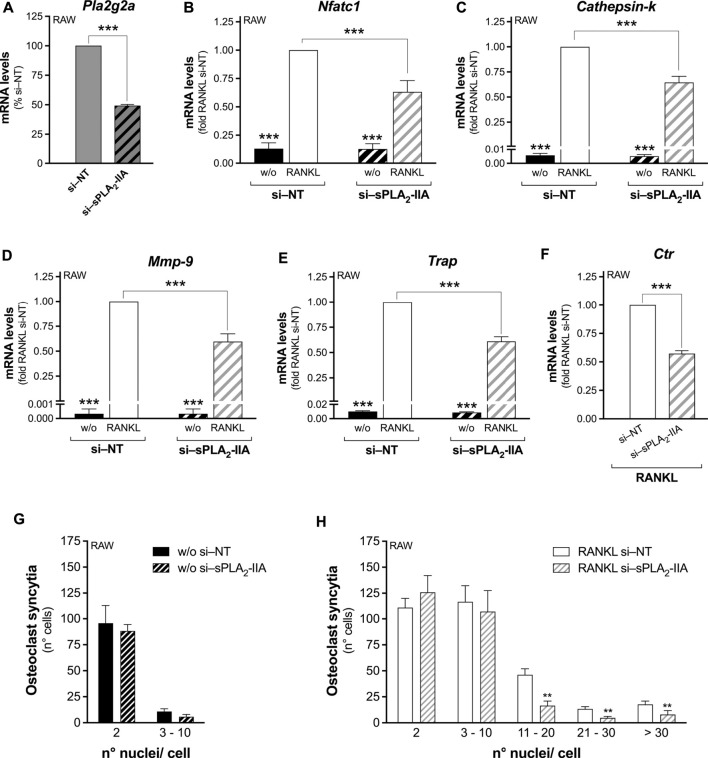
Secreted PLA_2_-IIA interference in RAW264.7 precursor cells inhibits RANKL-induced osteoclast differentiation. RAW264.7 cells were interfered for 72 h with no-targeting (si–NT) or sPLA_2_-IIA specific (si–sPLA_2_-IIA) siRNAs, and subsequently treated in the absence (w/o) or presence of 15–30 ng/mL RANKL for the following 48 h. **(A)** *Pla2g2a* mRNA levels of 72-h interfered RAW264.7 cells were quantified by real-time qPCR, and normalized using *β2-microglobulin* expression, as the housekeeping gene. Data are means ± SEM from six independent experiments. ***p < 0.005, paired Student’s *t*-test. **(B–F)** After 48 h of RANKL treatment, the differentiation markers were quantified by real-time qPCR, and normalized using *β2-microglobulin* expression, as the housekeeping gene. The mRNA levels of *Ctr* were undetectable in undifferentiated cells. Data are expressed as fold of RANKL-differentiated si–NT cells (RANKL si–NT), and are shown as means ± SEM of three independent experiments. ***p < 0.005 versus RANKL si–NT (one-way ANOVA or paired Student’s *t*-tests). **(G,H)** Osteoclast syncytium formation was determined as number of nuclei/cell, by fluorescence microscopy. Data are means ± SEM of three independent experiments performed in duplicates. **p < 0.01 versus correspondent RANKL si–NT (paired Student’s *t*-tests).

The involvement of sPLA_2_-IIA was deepened using cells with reduced or abolished sPLA_2_-IIA expression. RAW264.7 macrophages were transfected with specific small interfering (si)RNAs that significantly decreased by 50% the sPLA_2_-IIA mRNA expression (Figure 1A). This treatment did not affect the mRNA levels of intracellular PLA_2_s, sPLA_2_-IIC and -IIE, but slightly reduced that of sPLA_2_-V (Supplementary Table S2). The RANKL-induced differentiation of siRNA transfected cells showed a significantly reduced transcription, by about 40%, of the main osteoclastogenesis markers (*Nfatc1*, *Cathepsin-k*, *Mmp-9*, *Trap* and *Ctr*), upon sPLA_2_-IIA interference (Figures 1B–F). The osteoclast-syncytium formation was also impaired by sPLA_2_-IIA interference, with lower numbers of osteoclasts with more than 10 nuclei, compared to cells interfered with the no-targeting siRNAs (Figures 1G,H).

**FIGURE 2:**
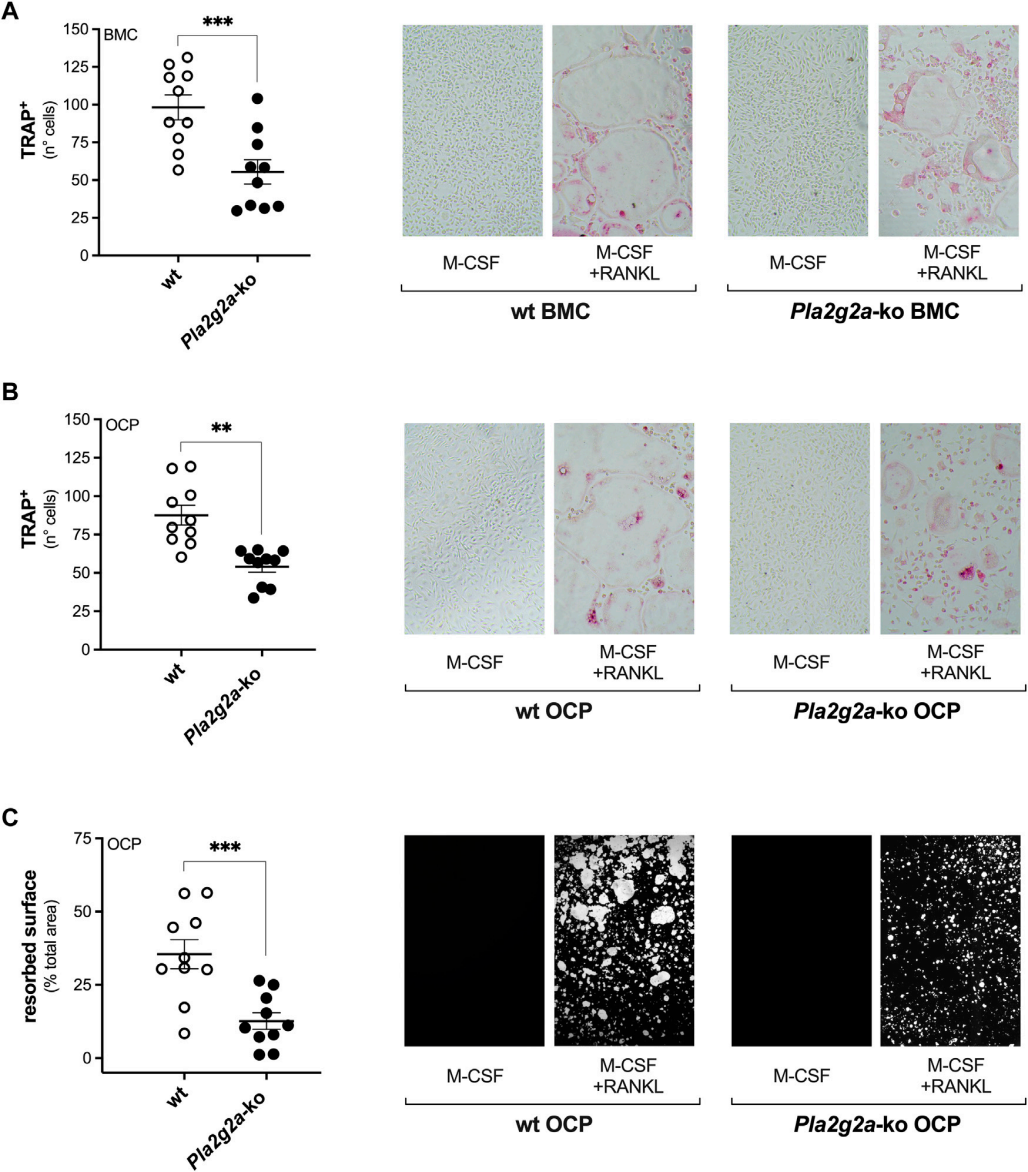
Osteoclast precursor cells derived from bone marrows of *Pla2g2a*-ko mice show reduced *ex-vivo* osteoclast differentiation and activity. **(A)** Freshly isolated total bone marrow cells (BMC) were differentiated with 20 ng/mL M-CSF and 5 ng/mL RANKL for 4 days, then fixed and stained for TRAP. **(B,C)** BMC were expanded with 40 ng/mL M-CSF for 4 days, and the obtained osteoclast precursor cells (OCP) were differentiated with 20 ng/mL M-CSF and 2.5 ng/mL RANKL for further 3–6 days. Then, OCP were fixed and stained for TRAP **(B)**, or their degrading activity was evaluated on Osteo Assay Surface plates **(C)**. Data are means ± SEM of ten age- and sex-matched mice for each genotype, analyzed in triplicates **(A,B)**, or duplicates **(C)**. On the right of each graph are representative images of TRAP staining **(A,B)**, and of Osteo Assay Surface plates analyzed for the degrading activity **(C)**, acquired with Nikon TMS-F inverted microscope, at a final magnification of ×5. **p < 0.01; ***p < 0.005 versus wt (unpaired Student’s *t*-tests).

Furthermore, *ex-vivo* osteoclast differentiation was carried out with primary osteoclast precursors obtained from bone marrow of wt and *Pla2g2a*-ko BALB/cJ mice (Balestrieri et al., 2006; Boilard et al., 2010). The PLA_2_ expression profile in M-CSF–expanded OCP from *Pla2g2a*-ko mice was similar to the wt counterpart, with the exception of *Pla2g1b*, *Pla2g2e* and *Pla2g5* that were expressed at higher levels, and the obvious lack of *Pla2g2a* (Table 1). Osteoclasts obtained from total bone marrow cells (BMC) or M-CSF–expanded OCP of *Pla2g2a*-ko mice, were less numerous (evaluated as TRAP-positive cells), and with lower degrading activity, compared to differentiated precursors from wt mice (Figure 2). In accordance with the RANKL-induced differentiation of sPLA_2_-IIA–interfered RAW264.7 macrophages, the differentiation of OCP from *Pla2g2a*-ko mice showed highly reduced marker transcription, and inhibition of osteoclast fusion, with lower numbers of osteoclasts containing more than 10 nuclei, compared to wt osteoclasts (Supplementary Figure S4).

Altogether, both pharmacological and molecular tools indicated the prevalent role of sPLA_2_-IIA in RANKL-induced differentiation of RAW264.7 macrophages. The impaired maturation, fusion and activity of osteoclasts derived from *Pla2g2a*-ko bone-marrow precursors paralleled the data obtained with the sPLA_2_-IIA–interfered RAW264.7 cell line, despite the increased transcription of *Pla2g2e* and *Pla2g5* in the *Pla2g2a*-ko OCP.

### 3.4 Pharmacological blockade of sPLA_2_-IIA impairs murine osteoclastogenesis

The crystal structures of sPLA_2_-IIA in complex with KH064 and LY311727 showed that these competitive inhibitors bind to the active site of sPLA_2_-IIA and also perturbate the binding surface at the entrance of the cavity (Lee et al., 2013). Therefore, these small molecules are able to block not only the sPLA_2_-IIA enzymatic activity, but also its possible interaction with binding partners. This was shown to be the case for methyl-indoxam, another active site sPLA_2_ inhibitor, which perturbates the interaction of various mouse sPLA_2_s to PLA_2_R1, shifting the affinity for this latter by up to 300-fold (Boilard et al., 2006).

**FIGURE 3:**
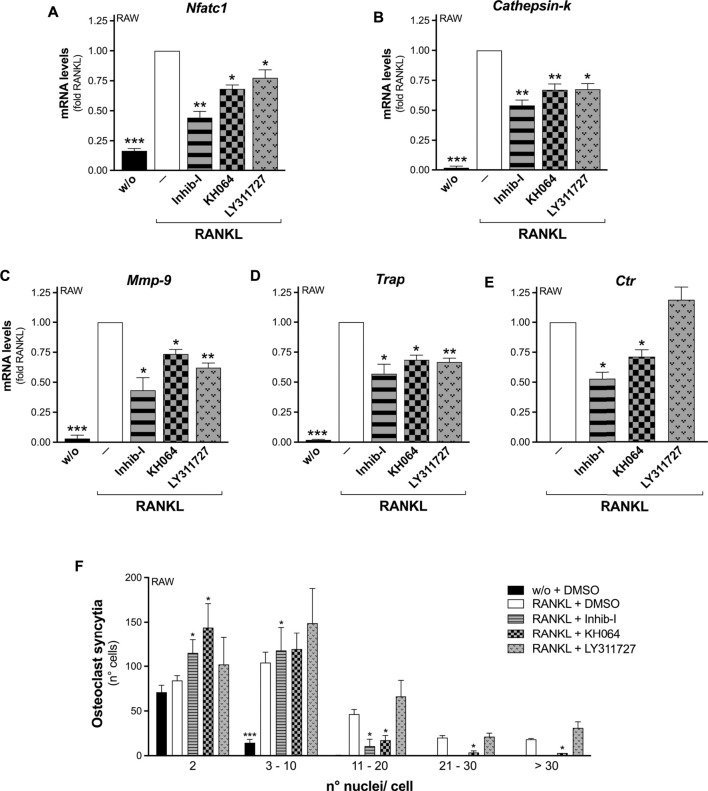
Bifunctional inhibitors of sPLA_2_-IIA impair RANKL-induced osteoclastogenesis of RAW264.7 macrophages. **(A–E)** RAW264.7 cells were treated without (w/o) or with 30 ng/mL RANKL for 72 h, in presence of the indicated sPLA_2_-IIA inhibitors (20 μM Inhib-I, 40 μM KH064, 30 μM LY311727) or with DMSO as carrier (‒). The differentiation markers were quantified by real-time qPCR, and normalized using *β2-microglobulin* expression, as the housekeeping gene. Data are expressed as fold of RANKL, and are means ± SEM of three independent experiments. **(F)** RAW264.7 cells were treated without (w/o) or with 15–30 ng/mL RANKL for 72–96 h, in presence of the above indicated sPLA_2_-IIA inhibitors or with DMSO as carrier. Osteoclast syncytium formation was determined as number of nuclei/cell, by fluorescence microscopy. Data are means ± SE of three independent experiments. *p < 0.05; **p < 0.01; ***p < 0.005 versus correspondent RANKL (paired Student’s *t*-tests).

Differentiation assays carried out in the presence of the small molecule KH064 showed that it acted similarly to the peptide Inhib-I, impairing RANKL-induced osteoclastogenesis. Indeed, both inhibitors significantly reduced the RANKL-stimulated transcription of all five osteoclastogenesis markers monitored, either with RAW264.7-macrophage precursors (Figures 3A–E), and with wt OCP (Supplementary Figures S5A–E). In addition, both inhibitors impaired osteoclast cell-to-cell fusion, as indicated by the decreased number of osteoclasts with more than 10 nuclei among RANKL-differentiated RAW264.7 cells (Figure 3F), and with more than 3 nuclei with wt OPC (Supplementary Figures S5F,G). As further confirmation, a significantly reduced formation of TRAP-positive cells from wt OCP was obtained in the presence of both KH064 and Inhib-I (Supplementary Figure S6). Strikingly, all these inhibitory effects were not seen differentiating *Pla2g2a*-ko OCP (Supplementary Figures S5, S6).

The LY311727 inhibitor was also capable to inhibit the osteoclastogenesis of RAW264.7 cells, but less potently, as it reduced RANKL-triggered *Nfatc1*, *Cathepsin-k*, *Mmp-9*, and *Trap* transcription, but not that of *Ctr*, nor the osteoclast-syncytium formation (Figure 3).

The results obtained with two structurally different inhibitors of sPLA_2_-IIA, namely Inhib-I and KH064, further support the involvement of this protein in the different steps of the murine osteoclastogenesis process, from the transcriptional remodeling of key genes to the osteoclast fusion. The absence of any effect with *Pla2g2a*-ko OPC confirmed the specificity of these inhibitors toward sPLA_2_-IIA, at least under our assay conditions.

### 3.5 Secreted PLA_2_-IIA regulates the osteoclastogenesis of RAW264.7 macrophages by two distinct mechanisms

The results shown so far provide the rationale to analyze in more detail the mechanism by which sPLA_2_-IIA regulates the osteoclastogenesis process. To this end, we took advantage of purified recombinant sPLA_2_-IIA of murine and human origin, including a H48Q catalytic site mutant which has less than 5% of wt activity (Edwards et al., 2002). The active site and catalytic mechanism of these two sPLA_2_-IIA orthologs are conserved and both enzymes share the same interfacial kinetic properties and substrate specificities (Cupillard et al., 1999; Valentin et al., 1999; Valentin et al., 2000; Singer et al., 2002). However, they have a rather low identity at the protein level (69%), especially on the protein surface, with a number of different amino acids that likely explain their different binding properties to proteins including PLA_2_R1 and Factor Xa (Cupillard et al., 1999; Mounier et al., 2000). Experimental evidence indicated that the 70–74 region of human sPLA_2_-IIA is relevant for sPLA_2_-IIA binding to membranes or putative signaling partners (Tseng et al., 1996). In addition, peptides derived from this sPLA_2_-IIA primary sequence were reported to competitively antagonize the sPLA_2_-IIA binding activity, and consequent sPLA_2_-IIA cellular functions, in a species-specific mode (Church et al., 2001).

**FIGURE 4:**
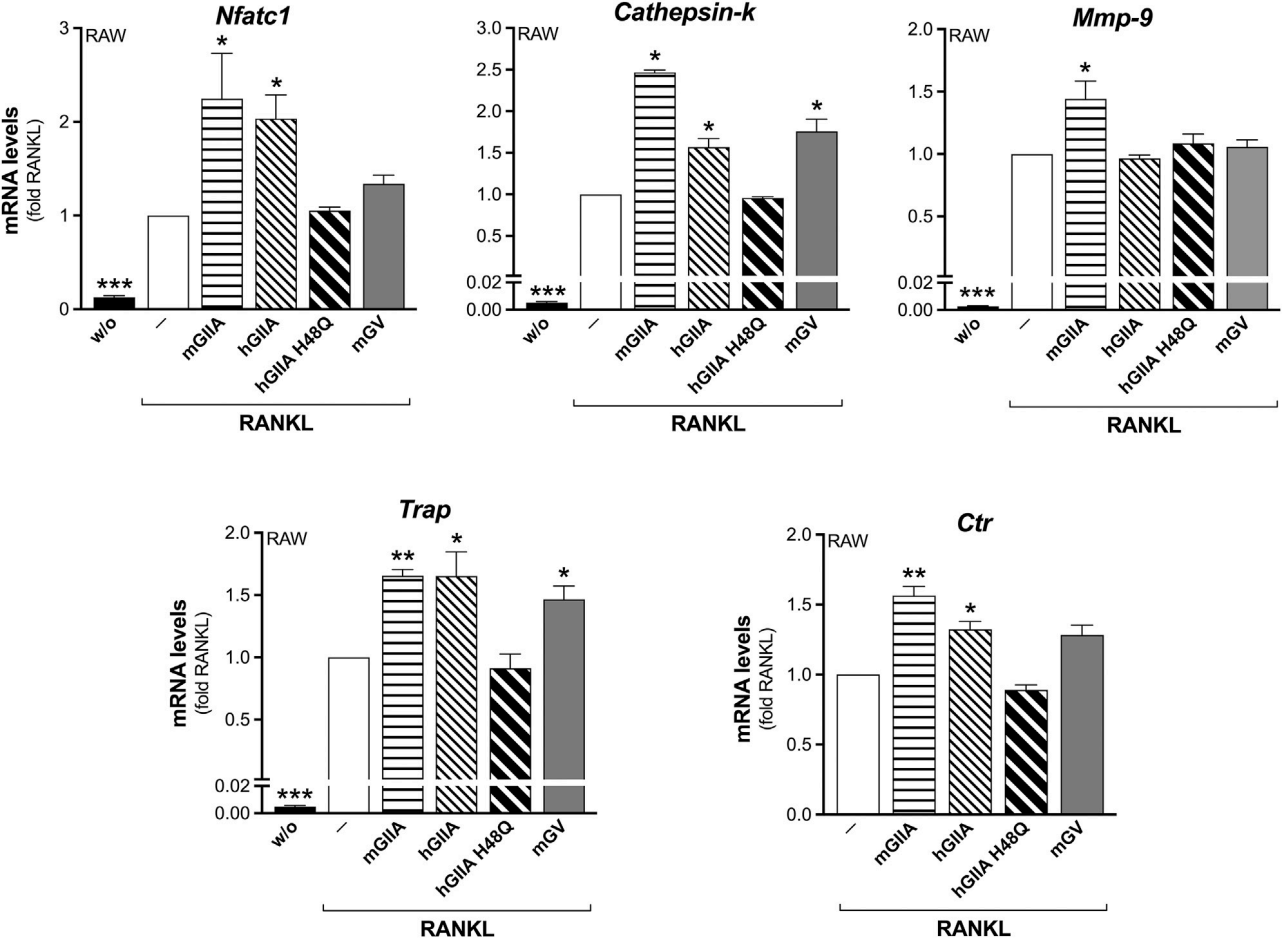
Murine recombinant sPLA_2_-IIA stimulates RANKL-induced osteoclastogenesis of RAW264.7 macrophages. RAW264.7 cells were treated without (w/o) or with 15 ng/mL RANKL for 72 h, in presence of the indicated recombinant proteins: 30 nM murine sPLA_2_-IIA (mGIIA), 30 nM human sPLA_2_-IIA (hGIIA), 30 nM human sPLA_2_-IIA H48Q (hGIIA H48Q), 100 nM murine sPLA_2_-V (mGV); or with PBS (‒) as carrier. The differentiation markers were quantified by real-time qPCR, and normalized using *β2-microglobulin* expression, as the housekeeping gene. Data are expressed as fold of RANKL, and are means ± SEM of three independent experiments. *p < 0.05; **p < 0.01; ***p < 0.005 versus RANKL (paired Student’s *t*-tests).

**FIGURE 5:**
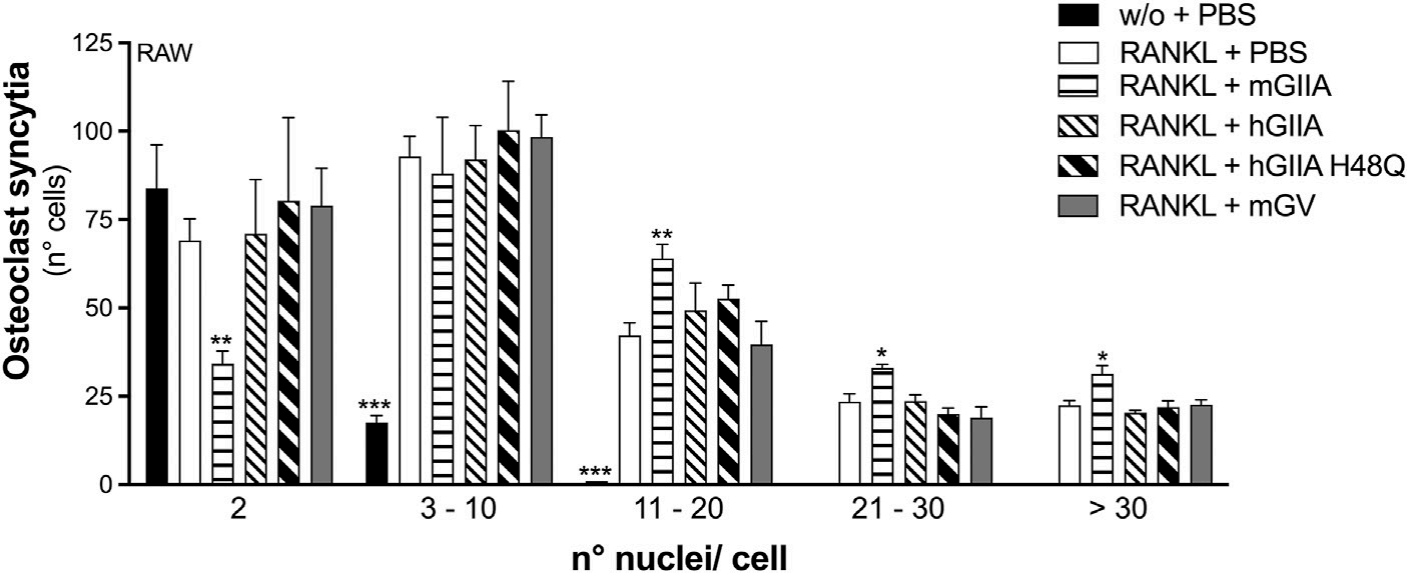
Murine recombinant sPLA_2_-IIA stimulates RANKL-induced osteoclast fusion of RAW264.7 macrophages. RAW264.7 cells were treated without (w/o) or with 15 ng/mL RANKL for 72 h, in presence of the indicated recombinant proteins: 30 nM murine sPLA_2_-IIA (mGIIA), 30 nM human sPLA_2_-IIA (hGIIA), 30 nM human sPLA_2_-IIA H48Q (hGIIA H48Q), 100 nM murine sPLA_2_-V (mGV); or with PBS as carrier. Osteoclast syncytium formation was determined as number of nuclei/cell, by fluorescence microscopy. Data are means ± SE of three independent experiments. *p < 0.05; **p < 0.01; ***p < 0.005 versus correspondent RANKL (paired Student’s *t*-tests).

The addition of recombinant mouse sPLA_2_-IIA (mGIIA) significantly reinforced the RANKL-induced transcription of all the differentiation markers analyzed, by more than 1.5-fold, during the differentiation of RAW264.7 macrophages (Figure 4). Even if with lower potency, the human enzyme (hGIIA) also increased the transcription of *Nfatc1*, *Cathepsin-k*, *Trap* and *Ctr*. Conversely, the catalytically inactive mutant H48Q (hGIIA H48Q) was without any effect (Figure 4). In parallel, the mouse sPLA_2_-V (mGV) was found to stimulate only the transcription of *Cathepsin-k* and *Trap*, but at a higher concentration of 100 nM (Figure 4). The *Mmp-9* transcription was stimulated only by mGIIA, and not by the human enzymes (active and mutated), nor by mGV (Figure 4). Finally, the RANKL-induced osteoclast syncytium formation was modulated only by mGIIA, with a significantly increased number of osteoclasts with more than 10 nuclei (Figure 5).

**FIGURE 6:**
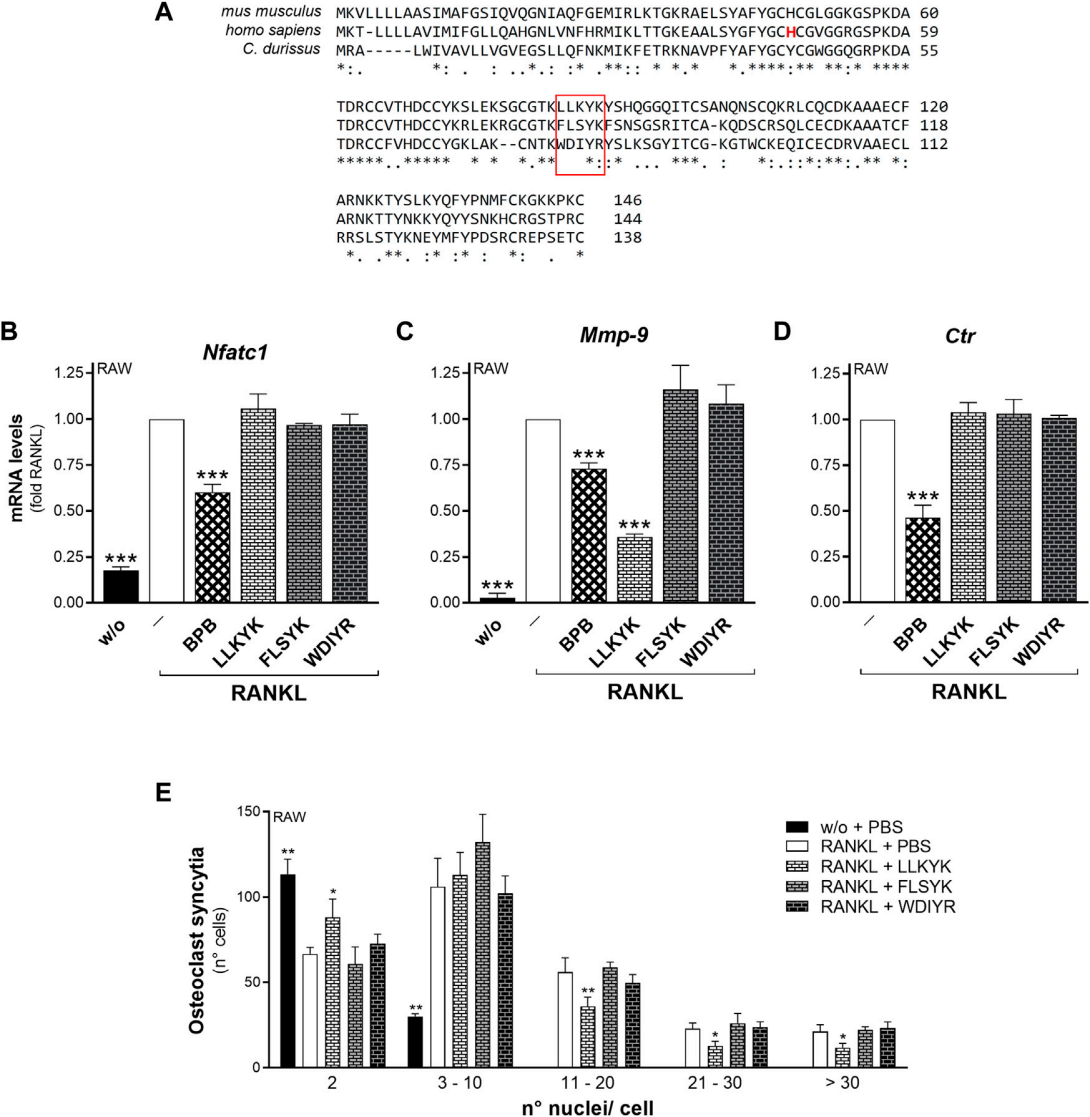
The LLKYK cyclic peptide inhibits RANKL-induced *Mmp-9* transcription and osteoclast fusion of RAW264.7 macrophages. **(A)** Alignment of sPLA_2_-IIA sequences from *mus musculus* (UniProtKB - P31482), *homo sapiens* (UniProtKB - P14555), and *C. durissus* (UniProtKB - P24027). Consensus symbols indicate residue conservation, as calculated with the Clustal Omega program. The pentapeptide sequences are boxed in red, and the catalytic histidine residue in the human sPLA_2_-IIA sequence is shown in red-letter code. **(B–D)** RAW264.7 cells were treated without (w/o) or with 15–30 ng/mL RANKL for 72 h, in presence of the carriers (‒), or 10 nM BPB, or 250 μM of the indicated peptides (LLKYK from the murine sPLA_2_-IIA sequence, FLSYK from that of human sPLA_2_-IIA, WDIYR from that of *C. durissus*). The differentiation markers were quantified by real-time qPCR analysis, and normalized using *β2-microglobulin* expression, as the housekeeping gene. Data are expressed as fold of RANKL, and are means ± SEM of at least three independent experiments. **(E)** RAW264.7 cells were treated without (w/o) or with of 15 ng/mL RANKL for 72 h, in presence of the indicated peptides (250 μM), or with PBS as carrier. Osteoclast syncytium formation was determined as number of nuclei/cell, by fluorescence microscopy. Data are means ± SE of six independent experiments. *p < 0.05; **p < 0.01; ***p < 0.005 versus correspondent RANKL (paired Student’s *t*-tests).

**FIGURE 7:**
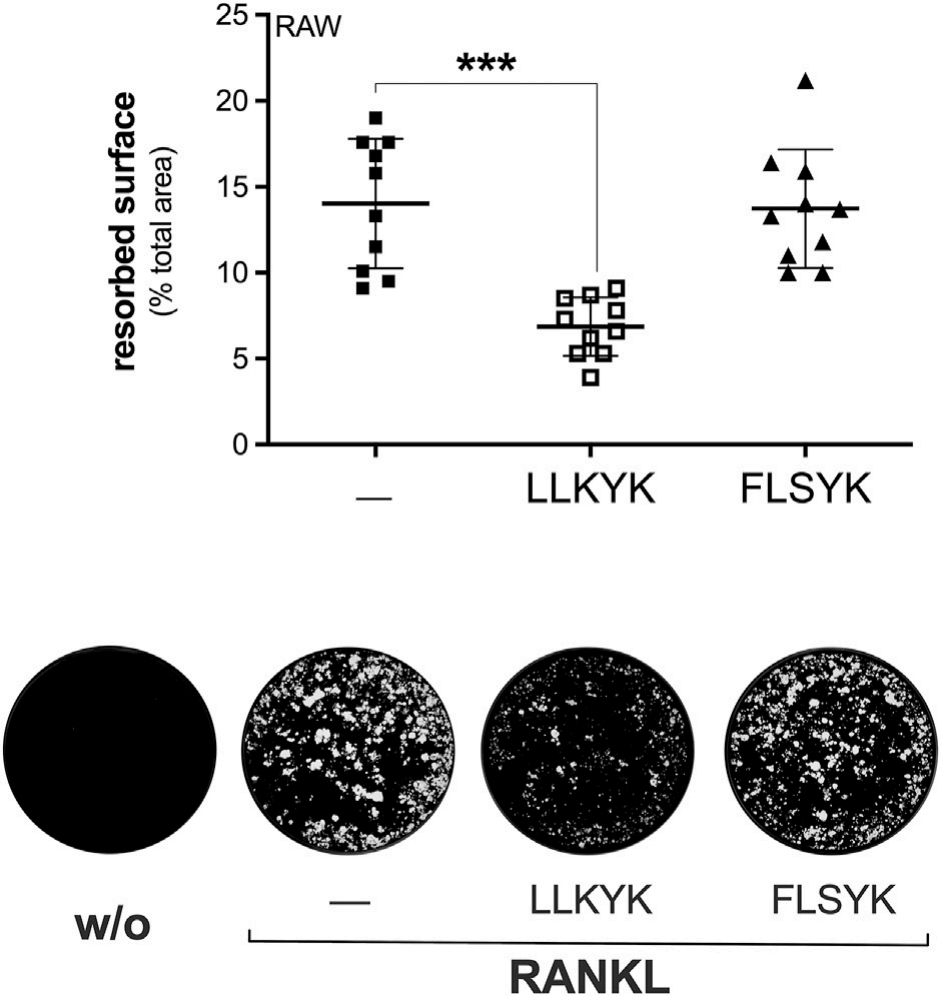
The LLKYK cyclic peptide inhibits RANKL-induced osteoclast resorbing activity of RAW264.7 macrophages. RAW264.7 cells plated on Osteo Assay Surface plates were treated without (w/o) or with 15 ng/mL RANKL for 7 days, in presence of PBS (‒) or 250 μM of the indicated cyclic pentapeptides (LLKYK from murine sPLA_2_-IIA sequence, and FLSYK from that of human sPLA_2_-IIA). Below are shown representative images of the degrading activity, acquired with the EVOS XL Core microscope at a final magnification of ×2. Data are means ± SEM of three independent experiments performed in triplicates. ***p < 0.005, paired Student’s *t*-test.

Three cyclic pentapeptides were synthesized based on the FLSYK sequence of human sPLA_2_-IIA (^70^FLSYK^74^; Church et al., 2001; Tseng et al., 1996), and the corresponding one in *C. durissus terrificus venom* sPLA_2_-IIA (^70^WDIYR^74^; Church et al., 2001; Tseng et al., 1996), and in mouse sPLA_2_-IIA (^84^LLKYK^89^) (Figure 6A). The LLKYK murine peptide, but not that from other species, was able to reduce the RANKL-induced *Mmp-9* transcription and cell-to-cell fusion, leaving unaffected the *Nfatc1* and *Ctr* transcription (Figures 6B–E). As the fusion step is crucial for the osteoclast activity, the effect of these peptides on the degrading activity was analyzed. The murine cyclic pentapeptide, LLKYK, was able to reduce by more than 50% the resorbing activity of mature osteoclasts on hydroxyapatite-coated plates, while the human FLSYK was ineffective (Figure 7).

In addition to the above experiments with the H48Q mutant, the role of mouse sPLA_2_-IIA enzymatic activity in osteoclastogenesis was confirmed by the use of BPB. This alkylating agent covalently binds to the histidine residue of the active site, but preserves the sPLA_2_-IIA surface properties and sPLA_2_-IIA binding properties to cells (Lee et al., 2013). Figure 6 shows that BPB treatment of RAW264.7 macrophages led to a clear inhibition of transcription for various RANKL-induced markers of the osteoclastogenesis, with reduction of *Nfatc1*, *Mmp-9* and *Ctr* levels by 40%, 30%, and 60%, respectively. In addition, *Cathepsin-k* and *Trap* transcription were significantly reduced by about 50% in RANKL-treated cells, and the osteoclast fusion process was not inhibited at all (Supplementary Figure S7).

Altogether, these results were indicative of the sole requirement of sPLA_2_-IIA catalytic activity for RANKL-induced marker transcription, with the exception of *Mmp-9*. Instead, sPLA_2_-IIA regulation of the osteoclast fusion occurred independently of its enzymatic activity, and was specific for mouse recombinant sPLA_2_-IIA.

### 3.6 Secreted PLA_2_-IIA regulates osteoclast fusion through p38 SAPK activation

**FIGURE 8:**
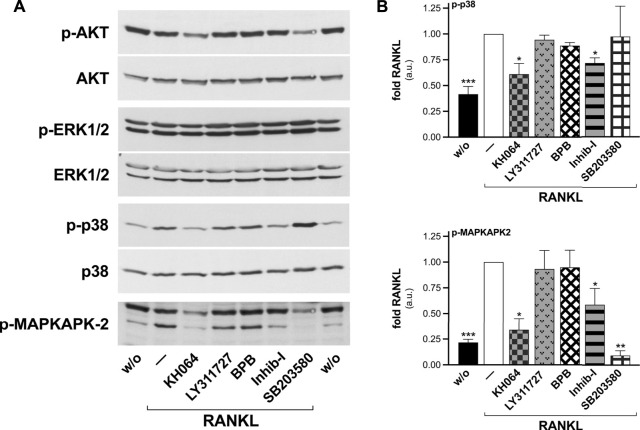
KH064 and Inhib-I counteract the RANKL-induced activation of p38 SAPK in differentiated RAW264.7 macrophages. RAW264.7 cells were treated without (w/o) or with 15–30 ng/mL RANKL for 72–96 h, in presence of the indicated sPLA2-IIA inhibitors (40 μM KH064, 30 μM LY311727, 10 nM BPB, 20 μM Inhib-I, 25 μM SB203580), or with DMSO as carrier (‒). **(A)** Western blotting of phosphorylated (p-AKT, p-ERK1/2, p-p38, p-MAPKAPK-2), and total AKT, ERK1/2, and p38 are shown, from an experiment representative of at least three independents. **(B)** Densitometric analysis by arbitrary units (a.u.) of p38 (top) and 50-kDa MAPKAPK-2 (bottom) phosphorylation levels, normalized for the correspondent protein levels. Data are expressed as fold of cells treated with RANKL, and are means ± SE of at least three independent experiments. *p < 0.05; **p < 0.01; ***p < 0.005 versus RANKL (paired Student’s *t*-tests).

The syncytium formation constitutes a crucial step for the osteoclast bone resorbing activity (Soe, 2020), and the unveiled catalysis-independent regulation of this process by sPLA_2_-IIA pushed toward the definition of the signaling pathways involved. Evaluation of RANKL downstream effectors in RAW264.7 macrophages, by Western blot analysis, displayed a significant activation of p38 SAPK and of one of its downstream substrates (MAPKAPK-2), while the phosphorylation levels of ERK1/2 MAPKs and AKT did not significantly differed from that of undifferentiated cells (Figure 8). Cell treatment with the different sPLA_2_-IIA inhibitors revealed that Inhib-I and KH064 counteracted the RANKL-induced phosphorylation of p38 SAPK and of MAPKAPK-2, while BPB and LY311727 were without effect (Figure 8).

**FIGURE 9:**
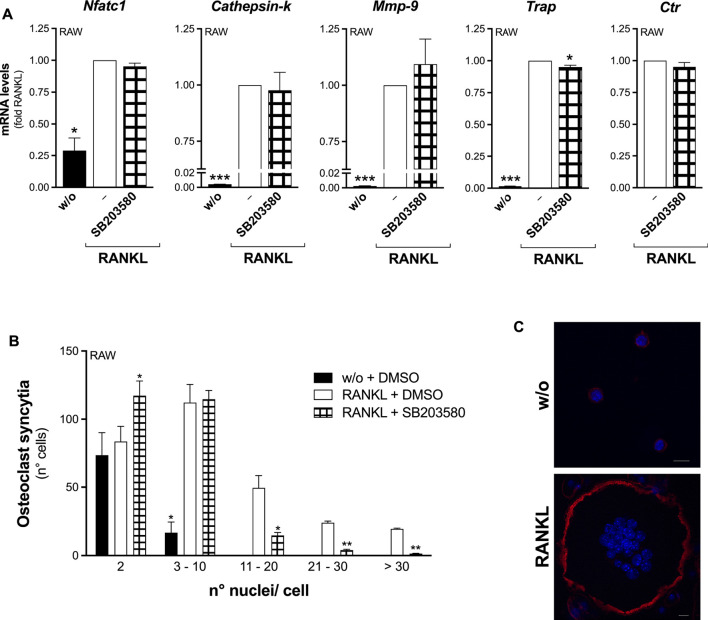
The p38 SAPK inhibitor selectively reduces RANKL-induced osteoclast fusion of RAW264.7 macrophages. **(A)** RAW264.7 cells were treated without (w/o) or with 15–30 ng/mL RANKL for 72 h, in presence of 25 μM SB203580 or with DMSO as carrier (‒). The differentiation markers were quantified by real-time qPCR, and normalized using *β2-microglobulin* expression, as the housekeeping gene. Data are expressed as fold of RANKL, and are means ± SEM of three independent experiments. **(B)** Osteoclast syncytium formation was determined as number of nuclei/cell, by fluorescence microscopy. Data are means ± SE of three independent experiments. *p < 0.05; **p < 0.01; ***p < 0.005 versus correspondent RANKL (paired Student’s *t*-tests). **(C)** Representative confocal images of RAW264.7 cells treated without (w/o) or with RANKL for 72 h, fixed and stained for confocal imaging. Phalloidin staining allows visualization of filamentous actin (red), while Hoechst staining reveals cell nuclei (blue, see Section 2 for details). Scale bars, 10 µm.

The osteoclastogenesis of RAW264.7 macrophages in the presence of p38 inhibition by SB203580, showed a foreseen blunted RANKL-induced phosphorylation of MAPKAPK-2 (Figure 8), but also a selectively impaired osteoclast fusion (Figure 9). Consistently, *ex-vivo* differentiation of M-CSF–expanded OPC, both wt and *Pla2g2a*-ko, upon SB203580 treatment produced significantly less TRAP-positive cells, maintaining unaffected the rate of the osteoclastogenesis marker transcription (Supplementary Figure S8).

These results suggested that p38 SAPK, a kinase part of the RANKL-triggered signaling (Park et al., 2017), mediated the osteoclast fusion but not the osteoclastogenesis marker transcription. In addition, p38 SAPK seemed to be downstream of the sPLA_2_-IIA catalysis-independent modulation of the osteoclastogenesis, selectively required for a fully functional osteoclast multinucleation.

## 4 Discussion

The findings here summarized prove the sPLA_2_-IIA multimodal regulation of RANKL-induced differentiation of murine osteoclast precursor cells. Whereas the osteoclastogenesis marker transcription requires sPLA_2_ hydrolytic activity, sPLA_2_-IIA participation in the osteoclast fusion and degrading activity occurs in a catalysis-independent manner and leads to the increased activation of p38 SAPK, presumably through the interaction with a still unidentified receptor.

This experimental evidence arises from *in-vitro* osteoclastogenesis assays performed with the well-validated and simplified model, based on the use of RAW264.7 macrophages as osteoclast precursor cells (Cuetara et al., 2006). The results obtained with this Abelson leukemia virus-transformed cell line, established from a BALB/c mouse strain (BAB/14; Raschke et al., 1978), were validated in *ex-vivo* osteoclastogenesis assays with primary precursor cells from BALB/cJ mice. Within these model systems, the transcriptional remodeling induced by RANKL results in increased sPLA_2_-IIA, -IIE, and -V mRNA levels, suggesting their involvement in the differentiation process or in mature osteoclast activity. These sPLA_2_s are all located in the distal part of the mouse chromosome 4 and lie within the so-called sPLA_2_ gene cluster (Valentin et al., 2000). The RANKL-triggered transcription of sPLA_2_-IIA could be consequent to an increased NF-κB activity, as this transcription factor is downstream the RANKL-activated signaling (Park et al., 2017), and has been reported to mediate the sPLA_2_-IIA transcription under inflammatory conditions. Indeed, cytokines such as TNF-α and IL-1β that are upregulated during inflammatory processes were shown to promote sPLA_2_-IIA expression in different cellular lineages, through the involvement of the transcription factor NF-κB (Vadas et al., 1991; Couturier et al., 1999; Beck et al., 2003). Though unable to detect sPLA_2_-IIA protein expression, with RAW264.7-cell lysates we succeeded in monitoring a PLA_2_ enzymatic activity that increased upon RANKL-induced differentiation, and that was impaired by inhibitors and a blocking antibody specific for sPLA_2_-IIA. This observation, together with the reported evidence of sPLA_2_-IIA involvement in pathologies of the bone system, led us to investigate the role of this enzyme in the osteoclastogenesis process.

The unique genetic background of inbred BALB/cJ mice allowed the production of a *Pla2g2a* knock out from 129Sv mice (Balestrieri et al., 2006; Boilard et al., 2010). Indeed, a number of mouse strains generally used for research purposes (C57BL/6, 129Sv, A/J, C58/J, P/J, and B10. RIII) are naturally knock out for *Pla2g2a*, due to a frameshift mutation in the gene (Kennedy et al., 1995; MacPhee et al., 1995). Here, both *Pla2g2a*-ko primary cells and sPLA_2_-IIA–interfered RAW264.7 macrophages were instrumental to unveil sPLA_2_-IIA role in both osteoclast maturation and fusion. In addition, the use of inhibitors selective for the different sPLA_2_-IIA activities dissected the underlying mechanisms of action.

The first open question that remains to be answered is how sPLA_2_-IIA could regulate osteoclast maturation via its enzymatic activity in a cell-autonomous context. The preferential cellular substrates for this enzyme are prevalently distributed in the inner (cytosolic) leaflet of normal plasma membranes (as phosphatidylserine, phosphatidylethanolamine or phosphatidylglycerol), and are thus not accessible in resting cells, also because the required conditions for sPLA_2_-IIA hydrolysis are not met in the cytosol (Bezzine et al., 2002; Singer et al., 2002). Of note, phosphatidylserine externalization has been reported to occur in pre-osteoclasts obtained from mouse bone marrow-derived cells, and was shown to be crucial for their M-CSF/RANKL-induced multinucleation (Kang et al., 2020). Interestingly, we observed an increased transcription of TMEM16F, a lipid scramblase and major contributor to the process of phosphatidylserine exposure (Pedemonte and Galietta, 2014), during the first 24 h of RANKL-induced differentiation of RAW264.7 macrophages (data not shown). A hallmark of TMEM16 scramblases is their lack of specificity, as phosphatidylinositol transbilayer movement across proteoliposomes reconstituted with purified fungal *Nectria haematococca* TMEM16 has been reported (Wang et al., 2018). Among the metabolites mediating sPLA_2_-IIA cellular effects, the lysophosphatidylinositol could be a reasonable candidate, as several studies have already highlighted its involvement in the osteoclastogenesis process (Whyte et al., 2009; Mosca et al., 2021). In particular, we have recently showed that lysophosphatidylinositol stimulates the osteoclast maturation of RAW264.7 macrophages, with increased transcription of *Nfatc1, Cathepsin-k, Trap, and Ctr*, without any effect on the osteoclast syncytium formation, through GPR55-mediated signal transduction (Mosca et al., 2021). This is consistent with the requirement of sPLA_2_-IIA catalytic activity exclusively for the regulation of the osteoclastogenesis marker transcription in our assay conditions. Indeed, both murine and human recombinant sPLA_2_-IIA increased the RANKL-induced transcription of *Nfatc1, Cathepsin-k, Trap, and Ctr*, but not the catalytically inactive sPLA_2_-IIA H48Q mutant. In addition, treatment of RAW264.7 macrophages with the alkylating agent BPB, which halts the sPLA_2_-IIA enzymatic activity without changing the overall conformation of the protein surface (Lee et al., 2013), induced a significant reduction of the osteoclastogenesis marker transcription, without affecting the osteoclast fusion. Although this inhibitor is a general alkylating agent and its administration to intact cells cannot guarantee for a sPLA_2_-IIA specific targeting (Scott et al., 2021), we verified that under BPB treatment the sPLA_2_-IIA enzymatic activity is significantly impaired in RANKL-differentiated RAW264.7 macrophages (Table 2). However, off-target effects cannot be completely ruled out.

Substantial progress has been made recently in the identification of the molecular machinery involved in osteoclast fusion, but the role of the lipid components in osteoclast syncytium formation is still under investigation. In different cell-to-cell fusion models, changes in membrane lipid composition have been shown to have relevant roles through the involvement of phospholipase activity (Chernomordik and Kozlov, 2008). For instance, in sperm fertilization of oocytes, changes in the acyl-chain composition of the main membrane glycerophospholipids may depend on another secreted phospholipase A_2_: sPLA_2_-X (Escoffier et al., 2010). Inhibition of osteoclast functions by sPLA_2_-IIA blockade has been reported by different groups, both *in vitro* and in animal models. In Pagetic bone samples, sPLA_2_-IIA was shown to be expressed at high levels and to stimulate the osteoclast differentiation and function by a mechanism that may be independent of its enzymatic activity (Allard-Chamard et al., 2014b). Consistently, under our assay conditions, sPLA_2_-IIA regulation of osteoclast cell-to-cell fusion seems to occur independently of its enzymatic activity. Indeed, not only two specific sPLA_2_-IIA inhibitors (Inhib-I and KH064), but mostly the cyclic pentapetide (LLKYK) derived from the murine sPLA_2_-IIA primary sequence, were able to impair the RANKL-induced syncytium formation and the osteoclast functional activity. Of note, the LLKYK peptide was not able to affect the osteoclastogenesis marker transcription, which seems to depend exclusively on sPLA_2_-IIA catalytic activity, with the exception of *Mmp-9* transcription that is regulated by sPLA_2_-IIA through multiple mechanisms. Differently from KH064, LY311727 did not affect the osteoclast fusion event. An explanation for the different behavior of these two sPLA_2_-IIA inhibitors in regulating the osteoclast fusion could reside in their peculiar ability to perturbate sPLA_2_-IIA interaction with a putative binding partner (Boilard et al., 2006), or their different cell-membrane permeability (Mounier et al., 2004; Jemel et al., 2011). Indeed, the sPLA_2_-IIA exhibits compartmentalization, not only for the enzymatic activity primarily occurring in the extracellular environment with a requirement of millimolar calcium concentrations. Even if the binding with a PLA_2_ receptor on the cell surface can occur, several other sPLA_2_-IIA interactions occur upon its internalization (Scott et al., 2021).

Studies over the last decade have revealed that dysregulated lipid metabolism is one of the fundamental metabolic alterations that enable cancer cell survival and sustain rapid tumor growth and cell proliferation (Kroemer and Pouyssegur, 2008). The expression of several sPLA_2_s is altered in various cancer cells, and in the neighboring stromal and immune cells at primary or metastatic tumor sites (Cummings, 2007; Murakami et al., 2011; Brglez et al., 2014; Sukocheva et al., 2019; Scott et al., 2021). Once secreted, sPLA_2_s can have both autocrine and paracrine roles, to induce metabolic and signaling changes in a tumor (Kamphorst et al., 2013). The expression of sPLA_2_-IIA is increased in the serum and tumors of patients with prostate, esophageal and lung cancers, and it is associated with poorer patient survival. Thus, the elevated levels of sPLA_2_-IIA in the plasma of these cancer patients has led to the suggestion of a biomarker role for sPLA_2_-IIA (Kupert et al., 2011). However, it remains unclear whether increased serum sPLA_2_-IIA levels are cancer-specific or whether they reflect inflammatory reactions during malignancy (Kupert et al., 2011). Of note, bone metastases are a common complication of epithelial cancers, of which breast, prostate and lung carcinomas are the most common (Kan et al., 2016). Serum levels of sPLA_2_-IIA can also rise from 100-fold to 1,000-fold during acute inflammatory disorders, such as sepsis, acute pancreatitis, peritonitis and chronic rheumatoid arthritis (Triggiani et al., 2005). Intriguingly, inflammatory pathologies such as rheumatoid arthritis and spondyloarthritides have also been associated with localized bone resorption and generalized osteoporosis (Szentpetery et al., 2017).

Many of the observed effects of sPLA_2_-IIA on biological systems, particularly in pathological settings characterized by persistent aberrant expression of this protein, may be attributed to direct or indirect perturbation of intracellular cell signaling pathways mediated by protein-protein interactions (Scott et al., 2021). Among the potential candidates, the M-type receptor PLA_2_R1 is able to bind murine sPLA_2_-IIA at low nanomolar concentrations, even if with an affinity 6–10-fold lower compared to sPLA_2_-IB (Cupillard et al., 1999; Rouault et al., 2007). In the lung, the proposed role for PLA_2_R1 is the clearance of PLA_2_-IB from the bronchoalveolar fluid by endocytosis and lysosomal degradation in airway smooth muscle cells (Tamaru et al., 2013). Otherwise, PLA_2_-IB binding to PLA_2_R1 may promote downstream signaling pathways, with activation of stress kinases and key enzymes part of the phospholipid and sphingolipid metabolism, able to generate pro-inflammatory bioactive lipid derivatives (Mandal et al., 2001). Furthermore, the highly basic nature of sPLA_2_-IIA allows its interaction with the negatively-charged heparan sulfates found at the cell surface (Murakami et al., 1999; Boilard et al., 2003), and with components of the coagulation cascade such as Factors Va and Xa (Mounier et al., 2000). The sPLA_2_-IIA interfacial binding site has been reported to be relevant for vimentin binding at the surface of apoptotic human T cells (Boilard et al., 2003). Instead, the arginine residues 74 and 100 in the human sPLA_2_-IIA sequence are crucial for the interaction with integrins α_V_β_3_ and *α*_4_β_1_ that leads to integrin activation and monocytic cell proliferation (Takada and Fujita, 2017). Intriguingly, several of these sPLA_2_-IIA binding partners are not only main components of the osteoclast adhesion zone, but play a crucial role in the activation of osteoclasts to resorb the bone, as it is the case for vimentin and integrins (α_V_β_3_, α_V_β_5_, and *α*_2_β_1_, among others) (Marchisio et al., 1984; Karanth et al., 2021).

More work will be necessary to dissect out each of the many regulatory pathways responsible for sPLA_2_-IIA actions during the osteoclast fusion and to identify the putative sPLA_2_-IIA receptor involved. These studies could pave the way for therapeutic application of sPLA_2_-IIA inhibitors. An ideal blocker would be required to selectively inhibit sPLA_2_-IIA binding to the specific receptor implicated in the osteoclast fusion. This will probably guarantee a tissue and cellular specific action. Several PLA_2_ inhibitors, including sPLA_2_ blockers, have been evaluated in clinical trials for the treatment of osteoarthritis, rheumatoid arthritis, atherosclerosis, sepsis and atopic dermatitis, among others, but none has reached the market (Nikolaou et al., 2019). The main limitation seems to be a lack of selectivity among the different human sPLA_2_s upon systemic administration, also as a consequence of a lack of a comprehensive knowledge on cellular functions connected with each sPLA_2_ subtype. Despite the fact that the rational design of selective inhibitors for the enzymatic activity of the different PLA_2_s is a challenging issue, due to the highly conserved catalytic domain within this family of enzymes (Lambeau and Gelb, 2008; Oslund et al., 2008; Burke and Dennis, 2009; Cao et al., 2013), selective targeting of sPLA_2_ ligand function has higher potentialities. This expectation could be fulfilled by monoclonal antibodies specifically raised against each enzyme (Pothlichet et al., 2020). Such sPLA_2_-interaction inhibitors could be proposed as alternatives to the bisphosphonates (Center et al., 2020; D'Oronzo et al., 2021), the actual treatment of choice for several osteoclast disorders, since bisphosphonates are contraindicated in older patients with kidney impairment or cardiac disease (Dos Santos Ferreira et al., 2017; Rogers et al., 2020; de Roij van Zuijdewijn et al., 2021).

## Data Availability

The raw data supporting the conclusions of this article will be made available by the authors, without undue reservation.
