# Supplementary material for: Multimodal regulation of the osteoclastogenesis process by secreted group IIA phospholipase A2
Source: Front Cell Dev Biol. 2022 Aug 29;10:966950. doi: 10.3389/fcell.2022.966950 (PMC9467450; doi:10.3389/fcell.2022.966950)

Supplementary Material

**Supplementary Table S1.** **Sequences of the real-time qPCR primers.**

| ***Genes*** | **Annealing T (°C)** | **Sequences** |
| --- | --- | --- |
| *β_2_-microglobulin* | 60 | Fw: 5'-TGGTGCTTGTCTCACTGACC-3'  Rv: 5'-GTATGTTGGCTTCCCATTC-3' |
| *Cathepsin-k* | 55 | Fw: 5'-CCTCTCTTGGTCTCCATACA-3'  Rv: 5'-ATCTCTCTGTACCCTCTGCA-3' |
| *Ctr* | 60 | Fw: 5'-ACCGACGAGCAACGCCTACGC-3'  Rv: 5'-GCCTTCACAGCCTTCAGGTAC-3' |
| *Mmp-9* | 55 | Fw: 5'-CTGTCCAGACCAAGGGTACAGCCT-3'  Rv: 5'-GTGGTATAGTGGGACACATAGTGG-3' |
| *Nfatc1* | 60 | Fw: 5'-CATGCAGCCATCATCGA-3'  Rv: 5'-TGGGATGTGAACTCGGAAGAC-3' |
| *Pla2g1b* | 62 | Fw: 5'-CCCCAGTGGACGACTTAGACA-3'  Rv: 5'-TCCAGCTTCTTGGCCTGACT-3' |
| *Pla2g2a* | 57 | Fw: 5'-TACAAGCGCCTGGAGAAAAG-3'  Rv: 5'-TTATCGCACTGACACA-3' |
| *Pla2g2c* | 66 | Fw: 5'-TTGCCATCTTCCTTGTCTTCATC-3'  Rv: 5'-CATCCTCTGGAACTGCCAGAA-3' |
| *Pla2g2d* | 66 | Fw: 5'-ATCTCCCAGGGCACTATCCA-3'  Rv: 5'-CCTCCTTGTCACAAGCACACA-3' |
| *Pla2g2e* | 62 | Fw: 5'-CCCAAGCTGGAAAAGTACCTCTT-3'  Rv: 5'-CGCTGGCAAGCCGTTCT-3' |
| *Pla2g2f* | 66 | Fw: 5'-TCAGGGCCTCTCCCTCTAAAA-3'  Rv: 5'-GGACTGCGATGGCAAAGAAT-3' |
| *Pla2g3* | 64 | Fw: 5'-TGGCCCAAAACATCAAAGTG-3'  Rv: 5'-GGCTTGATCTGGTGCTCACA-3' |
| *Pla2g4a* | 57 | Fw: 5'-CAGCTCTCAGGATTCCTTCGA-3'  Rv: 5'-TCATATATTCGTTCAAATTCATCTGGAT-3' |
| *Pla2g5* | 58 | Fw: 5'-CCCCAAGGATGGCACTGAT-3'  Rv: 5'-GCACAGTCTTTTTCCTCCAGTTG-3' |
| *Pla2g6* | 62 | Fw: 5'-TCCATGAGTACAATCAGGACATGA-3'  Rv: 5'-AGAAACGACTATGGAGAGTTTCTTCAC-3' |
| *Pla2g10* | 58 | Fw: 5'-CGATCTCCGATGGCTTACAT-3'  Rv: 5'-TTGGCATTTGTTCTCTGCTG-3' |
| *Pla2g12a* | 62 | Fw: 5'-CGCTCGGACTATCTCAGAACGT-3'  Rv: 5'-AAATGGATGACGCTGTCAAAGA-3' |
| *Pla2g12b* | 64 | Fw: 5'-CTGGGCTTTGTCTCCAACGT-3'  Rv: 5'-GGTCCACACGGTGTTGAACA-3' |
| *Pla2g16* | 57 | Fw: 5'-TACAGGCTGACCAGCGAGAACT-3'  Rv: 5'-CCACTCCAGCGATGCCTACCG-3' |
| *Trap* | 55 | Fw: 5'-AAATCACTCTTTAAGACCAG-3'  Rv: 5'-TTATTGAATAGCAGTGACAG-3' |

Fw, forward; Rv, reverse

**Supplementary Table S2. Modulation of PLA_2_ mRNA levels upon sPLA_2_-IIA interference in RAW264.7 macrophages.** RAW264.7 cells were interfered with si‒NT or si‒sPLA_2_-IIA siRNAs for 72 h (see Section 2 for details). Then, the mRNA levels of the indicated PLA_2_s were measured, by real-time qPCR, and expressed as fold of *si‒NT RAW264.7* *cells* (italic numbers). Subsequently, interfered cells were treated without (w/o) or with 30 ng/mL RANKL (RANKL) for further 48-72 h, and the PLA_2_ levels were quantified and expressed as fold of si‒NT cells treated without RANKL (w/o si‒NT).

|  | ***RAW264.7 cells*** | | **w/o** | | **RANKL** | |
| --- | --- | --- | --- | --- | --- | --- |
| **PLA_2_s** | *si‒NT* | *si‒sPLA_2_-IIA* | si‒NT | si‒sPLA_2_-IIA | si‒NT | si‒sPLA_2_-IIA |
| *Pla2g2a* | *1.00* | *0.48 ±0.01**** | 1.00 | 0.93 ±0.06 | 2.15 ±0.11 | 1.64 ±0.21^§^ |
| *Pla2g2c* | *1.00* | *1.04 ±0.05* | 1.00 | 1.11 ±0.08 | 2.94 ±0.38 | 3.36 ±0.55 |
| *Pla2g2e* | *1.00* | *1.11 ±0.04* | 1.00 | 1.24 ±0.15 | 4.28 ±1.00 | 4.89 ±0.31 |
| *Pla2g5* | *1.00* | *0.81 ±0.04** | 1.00 | 1.07 ±0.21 | 3.61 ±0.14 | 3.00 ±0.22^§^ |
|  |  |  |  |  |  |  |
| *Pla2g4a* | *1.00* | *1.13 ±0.02* | 1.00 | 1.00 ±0.07 | 0.63 ±0.18 | 0.71 ±0.09 |
| *Pla2g6* | *1.00* | *1.13 ±0.01* | 1.00 | 1.05 ±0.04 | 0.60 ±0.04 | 0.56 ±0.07 |

Data of interfered cells are expressed as fold of *si‒NT* *RAW264.7 cells*, and are means ±SEM of at least three independent experiments. **p* < 0.05; ****p* < 0.001 versus correspondent *si‒NT RAW264.7 cells,* by paired Student’s *t*-tests. Data of differentiated cells are expressed as fold of si-NT cells treated without RANKL (w/o si-NT), and are means ±SEM of at least three independent experiments. ^§^*p* < 0.05 versus correspondent si‒NT cells treated with RANKL (RANKL), by one-way ANOVA.

**
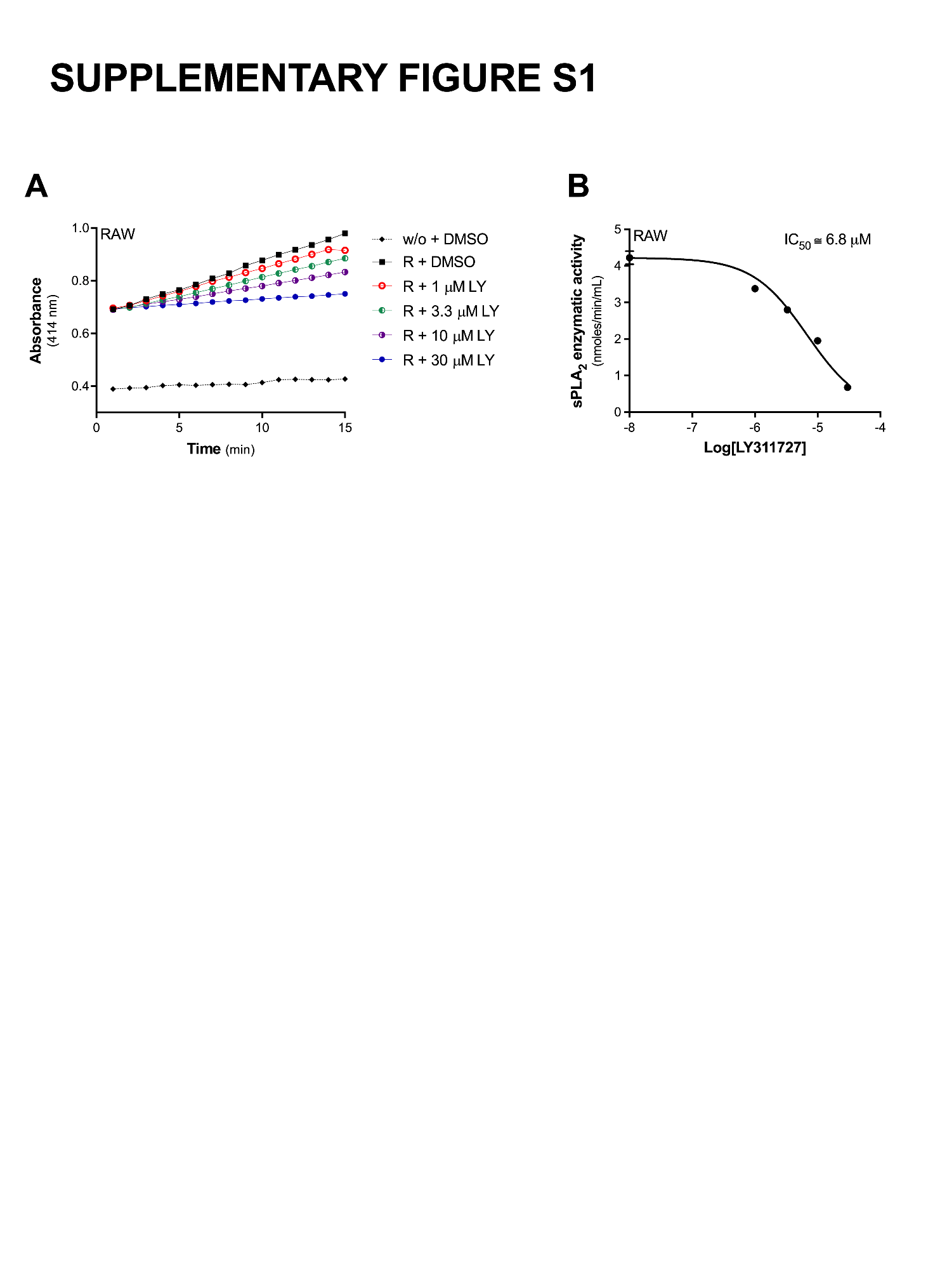
**

**Supplementary Figure S1. Secreted PLA_2_ activity measured in RAW264.7-cell lysates.** RAW264.7 cells were treated without (w/o) or with 15 ng/mL RANKL (R) for 72 h. Secreted PLA_2_ activity was measured in cell lysates (250 μg) in the presence of the indicated concentrations of LY311727 (LY) or with DMSO as vehicle (DMSO). **(A)** Absorbance-time graph of PLA_2_ activity. Data are means ± range of a representative experiment. **(B)** Dose-response curve of LY311727 inhibition of sPLA_2_ activity derived from the graph on the left, to extrapolate an apparent IC_50_ value.

**Supplementary Figure S2. Effects of PLA_2_ inhibitors on the osteoclast differentiation of RAW264.7 macrophages.** RAW264.7 cells were treated in the absence (w/o) or presence of 15-30 ng/mL RANKL for 72-96 h, with the indicated PLA_2_ inhibitors (20 μM Inhib-I, 1 μM BEL, 2 μM cPLA_2_α–Inh., 20 μM MAFP) or with DMSO as carrier (‒). The differentiation markers were quantified by real-time qPCR, and normalized using *β2-microglobulin* expression, as the housekeeping gene. Data are means ± SEM of five independent experiments. **p* < 0.05; ***p* < 0.01; ****p* < 0.005 versus RANKL (paired Student’s *t*-tests).


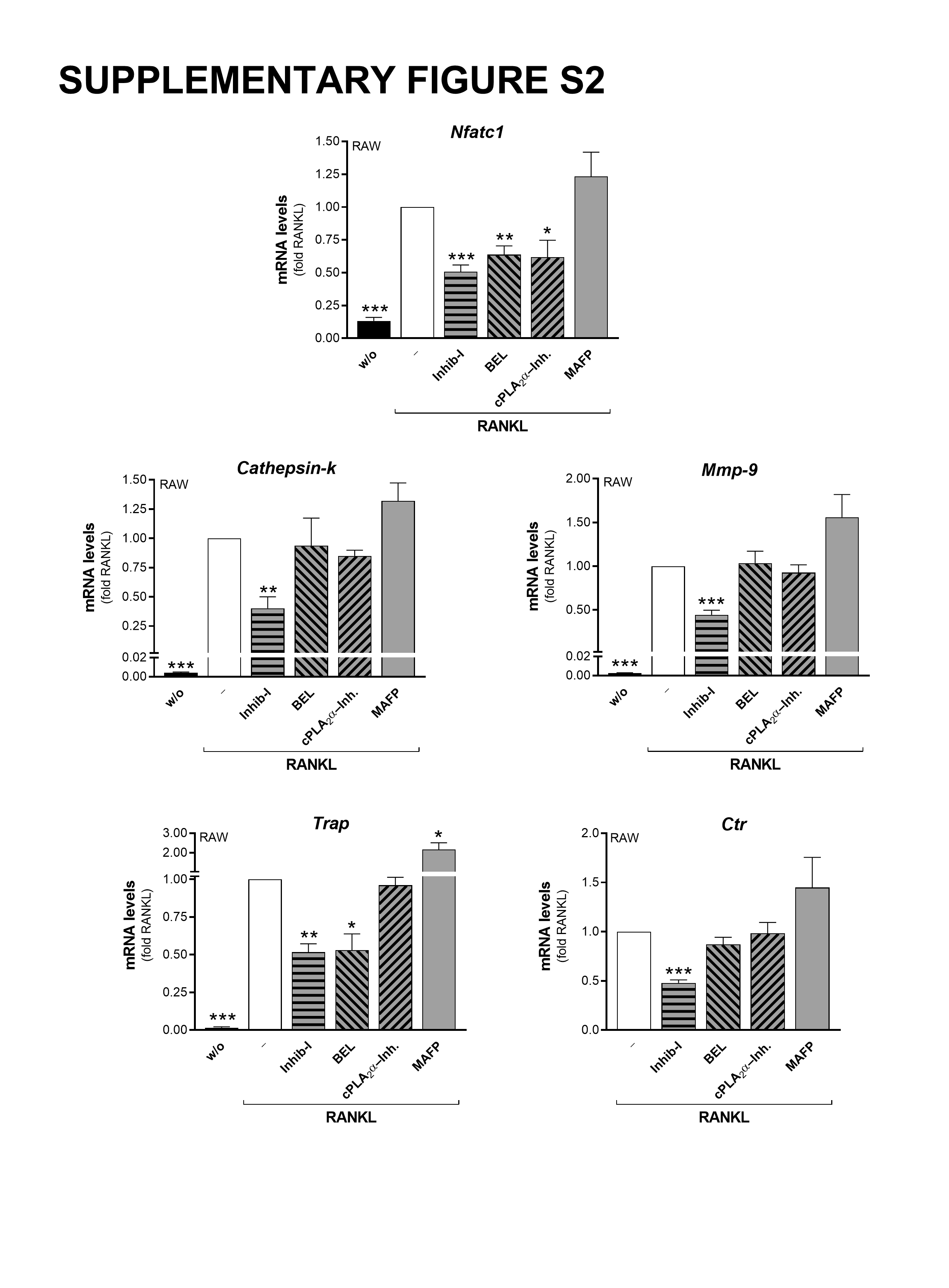


**Supplementary Figure S3. Effects of PLA_2_ inhibitors on the osteoclast fusion of RAW264.7 macrophages.** RAW264.7 cells were treated in the absence (w/o) or presence of 15-30 ng/mL RANKL for 72-96 h, with the indicated PLA_2_ inhibitors (20 μM Inhib-I, 1 μM BEL, 2 μM cPLA_2_α–Inh., 20 μM MAFP) or with DMSO as carrier. Osteoclast syncytium formation was determined as number of nuclei/cell, by fluorescence microscopy. Data are means ± SE of four independent experiments. **p* < 0.05; ***p* < 0.01 versus corresponding RANKL (paired Student’s *t*-tests).


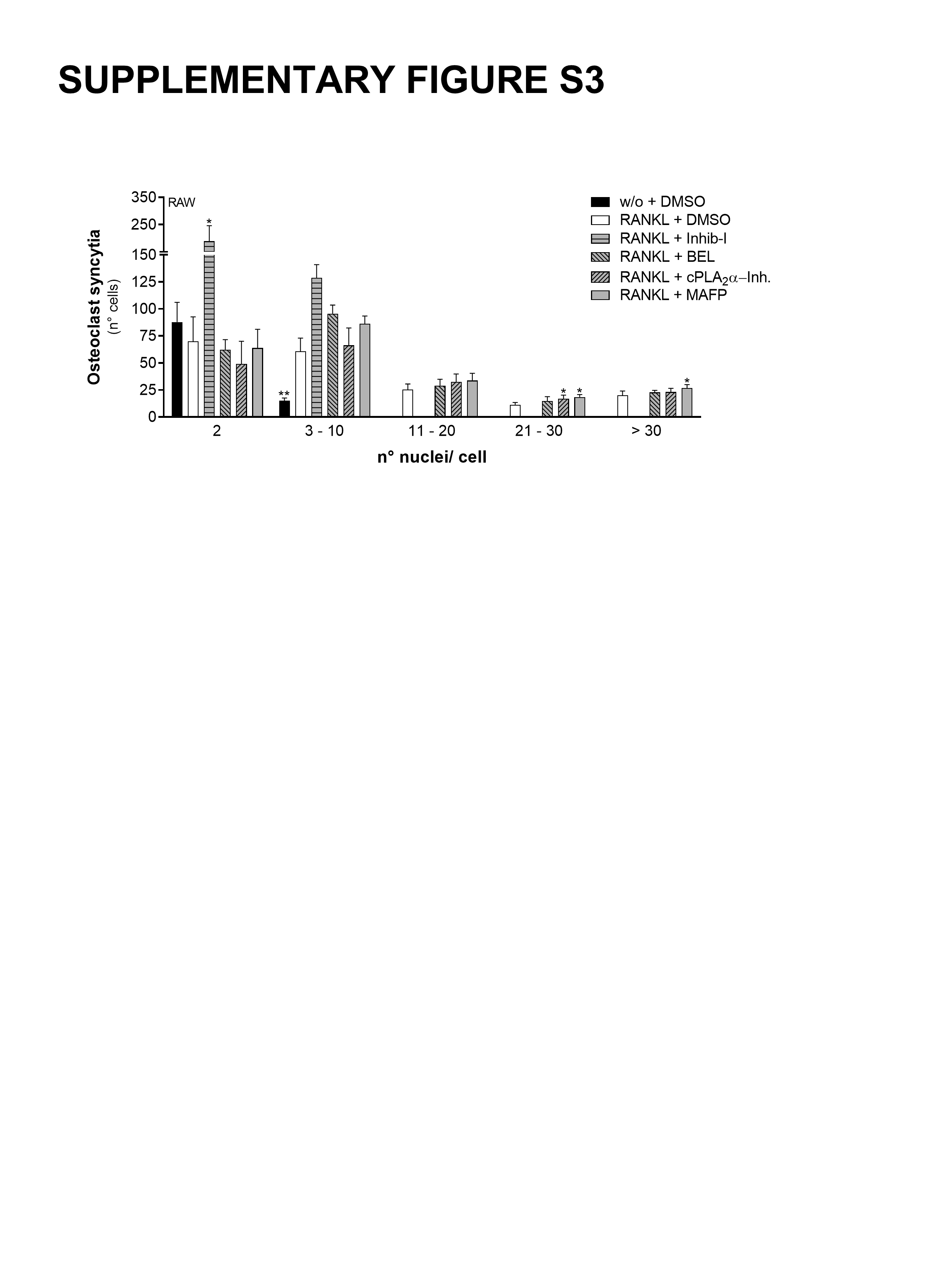


**Supplementary Figure S4. Osteoclasts from *Pla2g2a*-ko OCP show impaired *ex-vivo* osteoclastogenesis.** The M-CSF–expanded OCP were further treated with 20 ng/mL M-CSF alone (w/o) or with 20 ng/mL M-CSF and 2.5 ng/mL RANKL (RANKL) for 3-6 days for RNA extraction, or 4-5 days for immunofluorescence analysis. **(A)** The differentiation markers were quantified in RANKL-treated cells by real-time qPCR, and normalized using *β2-microglobulin* expression, as the housekeeping gene. Data are expressed as fold of differentiated wt OCP, and are shown as means ± SEM of ten age- and sex-matched mice for each genotype. **(B-C)** Osteoclast syncytium formation was determined as number of nuclei/cell, by fluorescence microscopy. Data are means ± SE of ten age- and sex-matched mice for each genotype. **p* < 0.05; ***p* < 0.01; ****p* < 0.005 versus correspondent RANKL wt (unpaired Student’s *t*-tests).


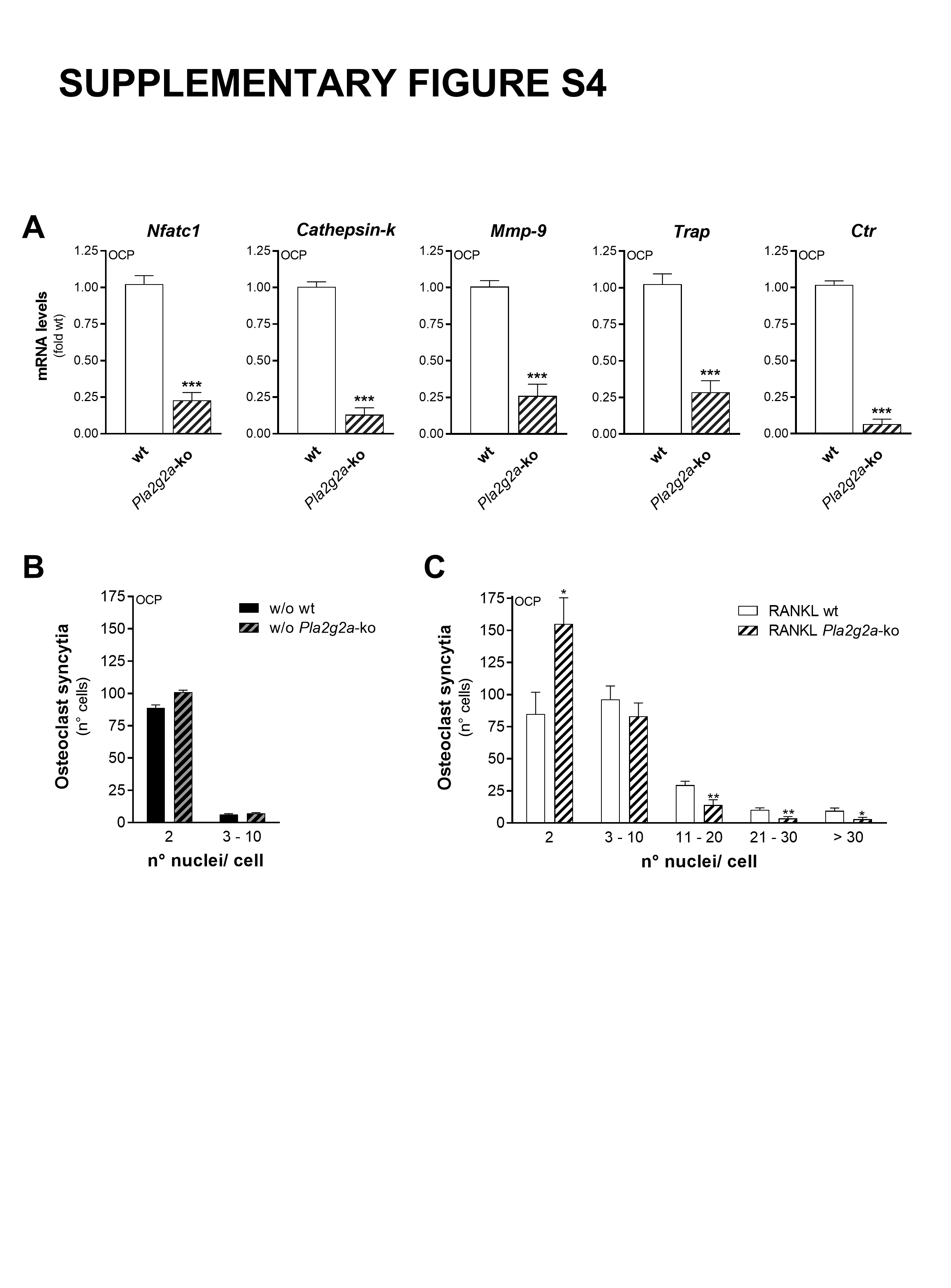


**Supplementary Figure S5. Inhibitors of sPLA_2_-IIA impair the RANKL-induced osteoclastogenesis of wt OCP.** The M-CSF–expanded OCP were further treated with 20 ng/mL M-CSF and 2.5 ng/mL RANKL, in presence of the indicated sPLA_2_-IIA inhibitors (20 μM Inhib-I; 40 μM KH064) or with DMSO as carrier (‒), for 6 days for RNA extraction, or 5 days for immunofluorescence analysis. **(A-E)** The differentiation markers were quantified by real-time qPCR, and normalized using *β2-microglobulin* expression, as the housekeeping gene. Data are expressed as fold of correspondent RANKL, and are means ± SEM of three age- and sex-matched mice for each genotype. The mRNA levels from RANKL *Pla2g2a*-ko, expressed as fold of RANKL wt, were: 0.09 ±0.01 for *Nfatc1*; 0.07 ±0.01 for *Cathepsin-k*; 0.12 ±0.03 for *Mmp-9*; 0.11 ±0.01 for *Trap*; 0.003 ±0.001 for *Ctr*. **(F-G)** Osteoclast syncytium formation was determined as number of nuclei/cell, under fluorescence microscopy. Data are means ± SE of three age- and sex-matched mice for each genotype. **p* < 0.05; ***p* < 0.01; ****p* < 0.005 versus RANKL wt (one-way ANOVA, **A-E**; or unpaired Student’s *t*-tests, **F-G**).


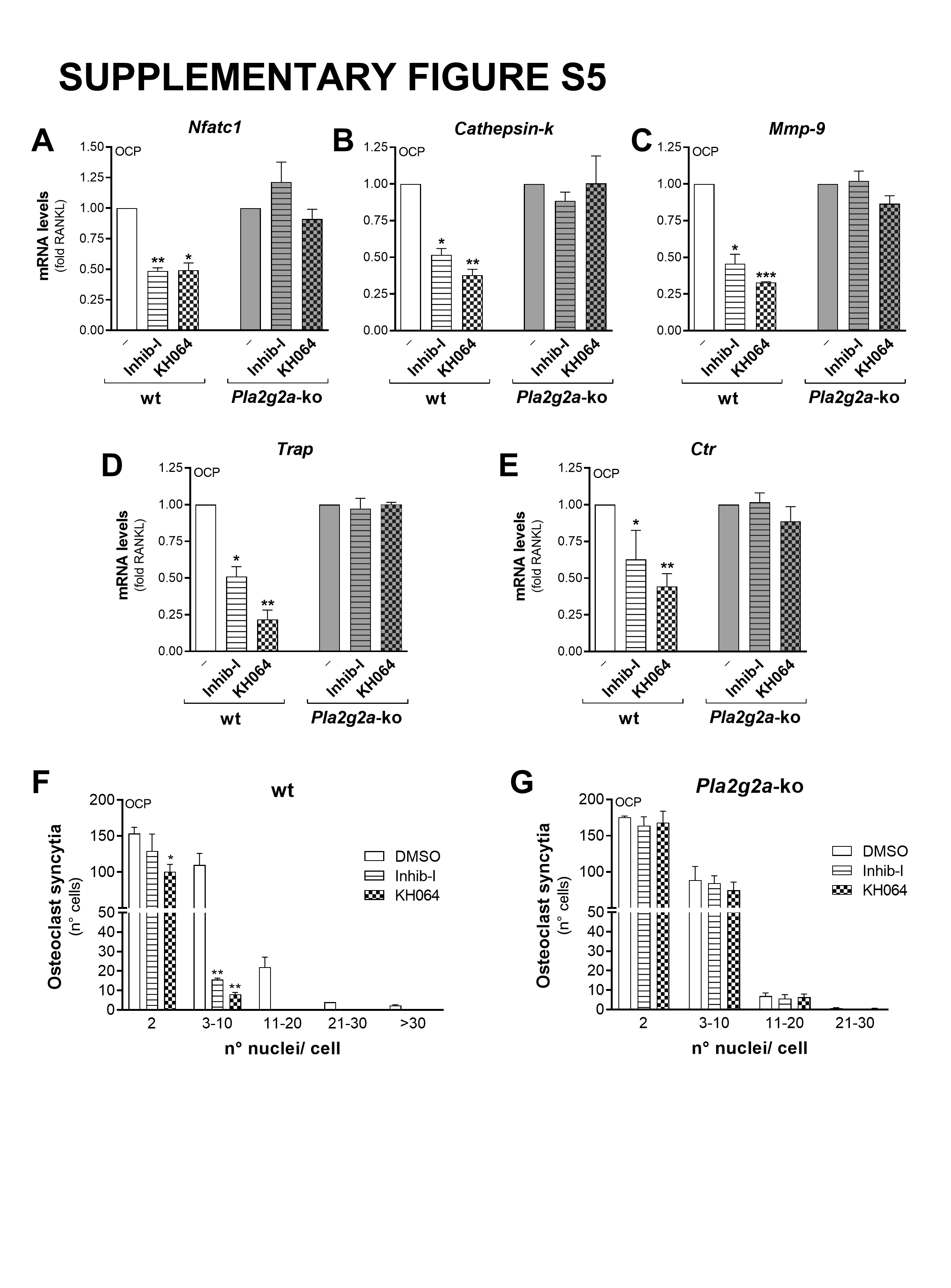


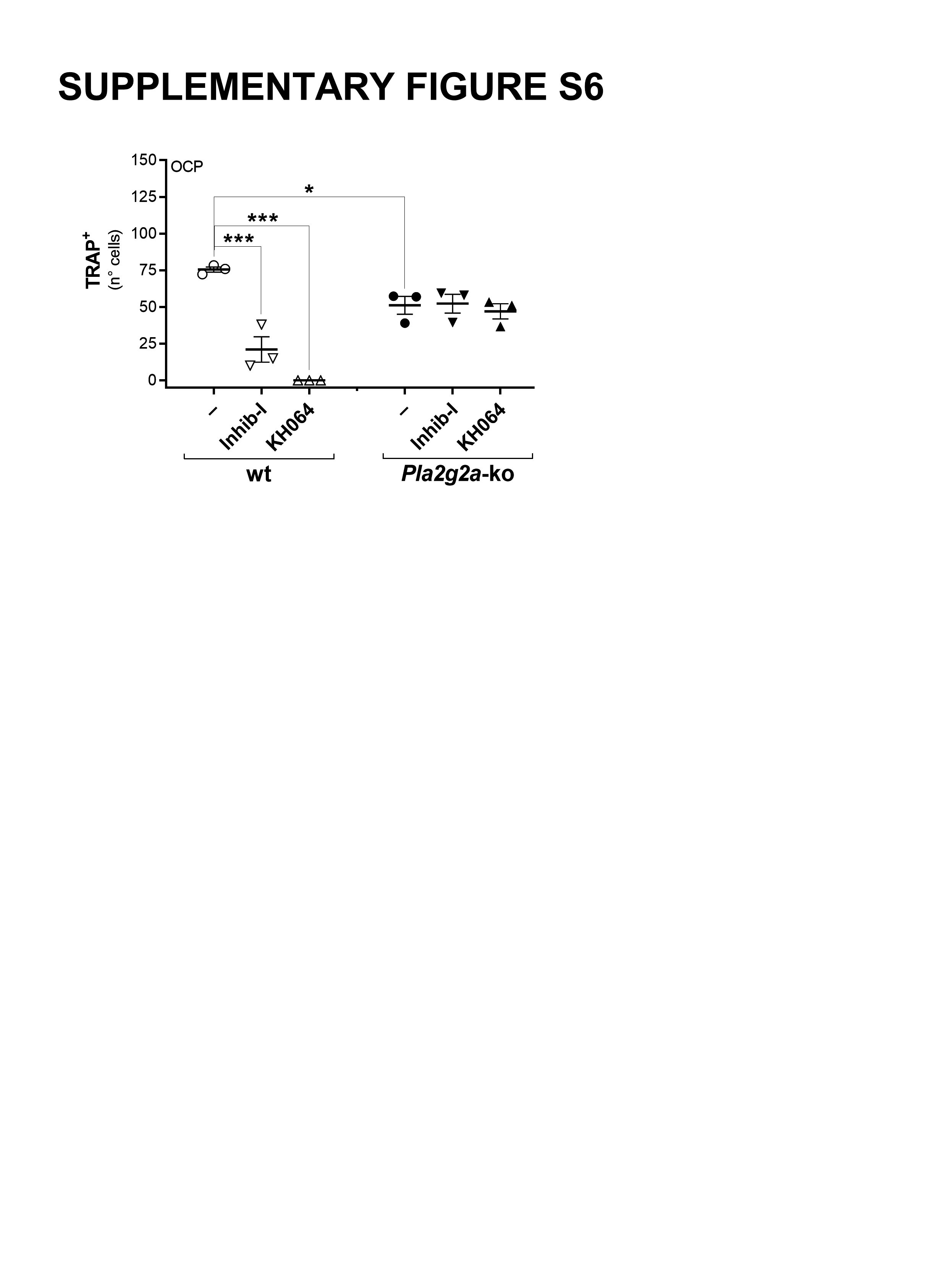


**Supplementary Figure S6. Inhibitors of sPLA_2_-IIA reduce the RANKL-induced formation of TRAP-positive osteoclasts from wt OPC.** The M-CSF–expanded OCP were further treated with 20 ng/mL M-CSF and 2.5 ng/mL RANKL, in presence of the indicated sPLA_2_-IIA inhibitors (20 μM Inhib-I; 40 μM KH064) or with DMSO as carrier (‒), for 5 days for TRAP staining. The quantification of TRAP-positive cells (TRAP^+^) is shown as means ± SEM of three age- and sex-matched mice for each genotype, analyzed in triplicates. **p* < 0.05; ****p* < 0.005 versus DMSO (‒) wt (one-way ANOVA).

**Supplementary Figure S7. BPB selectively inhibits RANKL-induced marker transcription of RAW264.7 macrophages.** RAW264.7 cells were treated without (w/o) or with of 30 ng/mL RANKL for 72 h, in presence of 10 nM BPB or with DMSO as carrier (‒). **(A-B)** The differentiation markers were quantified by real-time qPCR, and normalized using *β2-microglobulin* expression, as the housekeeping gene. Data are expressed as fold of RANKL, and are means ± SEM of six independent experiments. **(C)** Osteoclast syncytium formation was determined as number of nuclei/cell, by fluorescence microscopy. Data are means ± SE of four independent experiments. ***p* < 0.01; ****p* < 0.005 versus correspondent RANKL (paired Student’s *t*-tests).


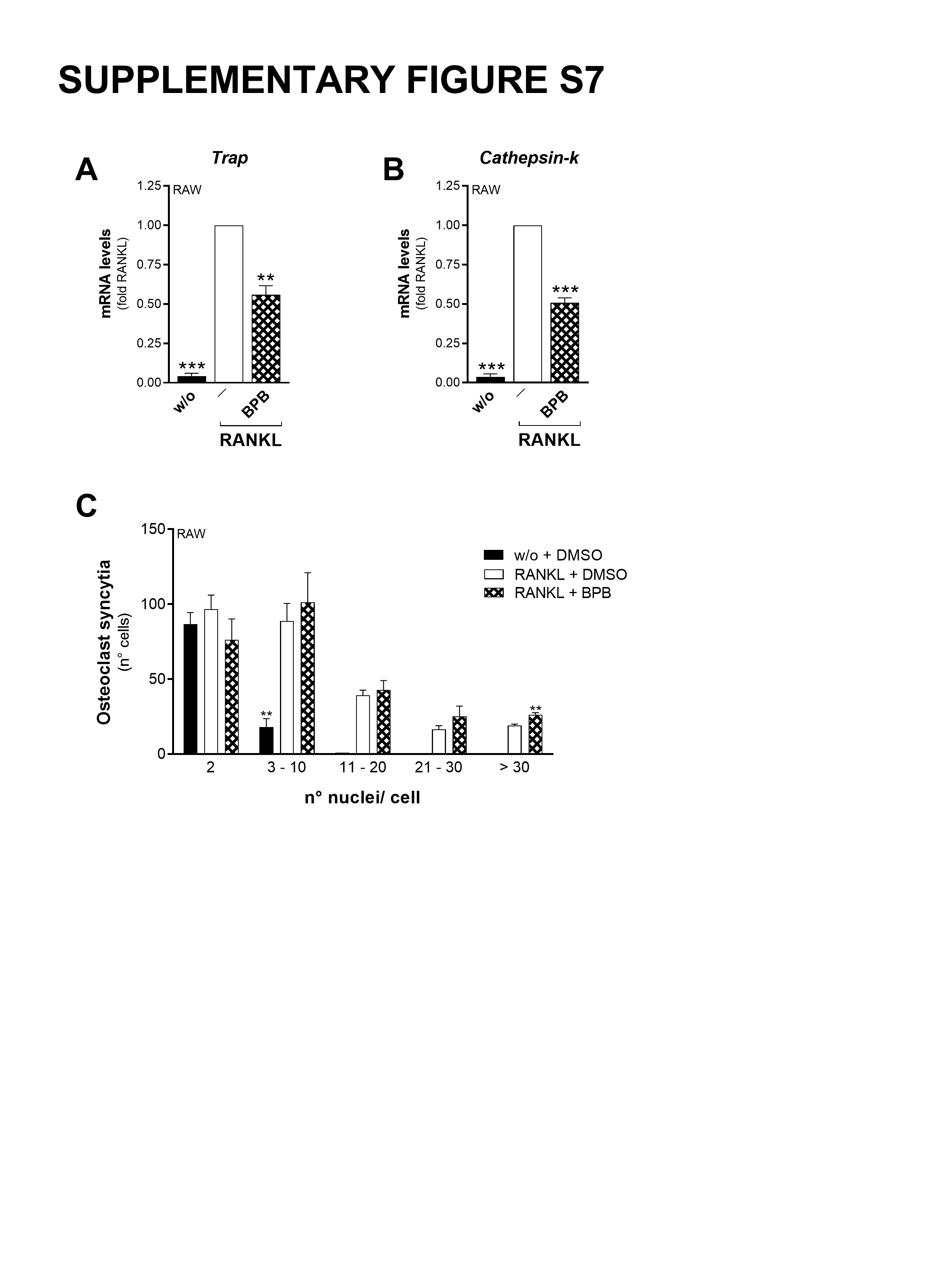


**Supplementary Figure S8. SB203580 selectively inhibits RANKL-induced osteoclast fusion of murine primary precursors.** The M-CSF–expanded OCP were further treated with 20 ng/mL M-CSF and 2.5 ng/mL RANKL, in presence of 1 µM SB203580 or with DMSO as carrier (‒), for 4 days for RNA extraction, or 6 days for TRAP staining. **(A-E)** The differentiation markers were quantified by real-time qPCR, and normalized using *β2-microglobulin* expression, as the housekeeping gene. Data are expressed as fold of correspondent RANKL, and are means ± SEM of three age- and sex-matched mice for each genotype. **(F)** The quantification of TRAP-positive cells (TRAP^+^) is shown as means ± SEM of five age- and sex-matched mice for each genotype, performed in triplicates. **p* < 0.05; ****p* < 0.005 versus correspondent DMSO (one-way ANOVA).


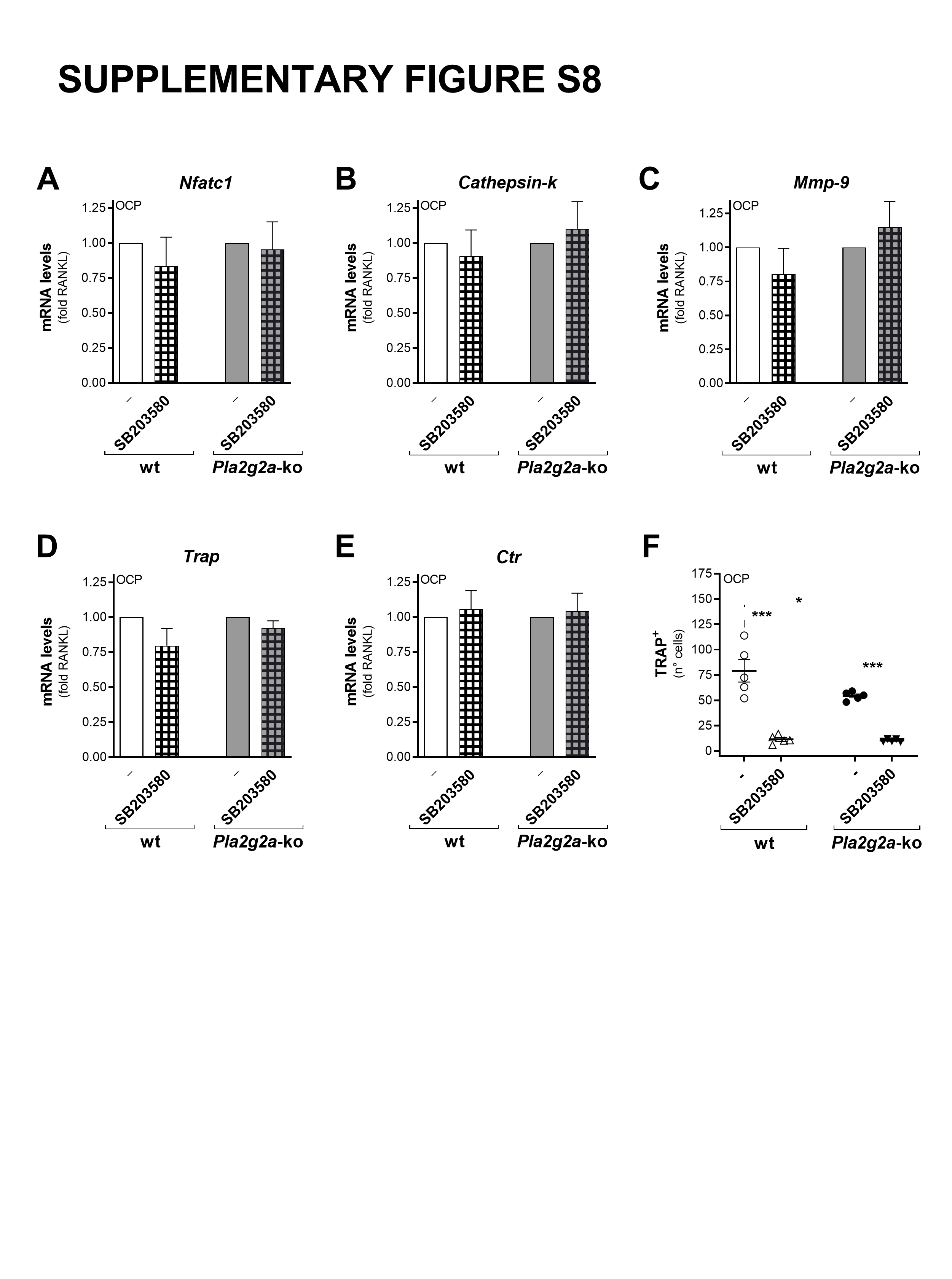

Supplement: Supplementary file 1 [file DataSheet1.docx]
